# A pH-sensitive CuHP composite hydrogel featuring antibacterial, antioxidant and osteogenic properties for treating diabetic periodontitis

**DOI:** 10.1093/rb/rbaf065

**Published:** 2025-06-23

**Authors:** Xianwen Lu, Sitong Hu, Zhaowenbin Zhang, Jing Bao, Bangping Cao, Jian Xie, Jiang Chang, Chen Yang, Xiaohong Wang, Jiansheng Su

**Affiliations:** Shanghai Engineering Research Center of Tooth Restoration and Regeneration & Tongji Research Institute of Stomatology & Department of Prosthodontics, Shanghai Tongji Stomatological Hospital and Dental School, Tongji University, Shanghai 200072, China; Zhejiang Engineering Research Center for Tissue Repair Materials, Wenzhou Institute, University of Chinese Academy of Sciences, Wenzhou 325000, China; Department of Orthodontics, School and Hospital of Stomatology, Liaoning Provincial Key Laboratory of Oral Disease, China Medical University, Shenyang 110002, China; Zhejiang Engineering Research Center for Tissue Repair Materials, Wenzhou Institute, University of Chinese Academy of Sciences, Wenzhou 325000, China; College of Biological Science and Medical Engineering, Donghua University, Shanghai 201620, China; Shanghai Engineering Research Center of Tooth Restoration and Regeneration & Tongji Research Institute of Stomatology & Department of Prosthodontics, Shanghai Tongji Stomatological Hospital and Dental School, Tongji University, Shanghai 200072, China; Shanghai Engineering Research Center of Tooth Restoration and Regeneration & Tongji Research Institute of Stomatology & Department of Prosthodontics, Shanghai Tongji Stomatological Hospital and Dental School, Tongji University, Shanghai 200072, China; Shanghai Engineering Research Center of Tooth Restoration and Regeneration & Tongji Research Institute of Stomatology & Department of Prosthodontics, Shanghai Tongji Stomatological Hospital and Dental School, Tongji University, Shanghai 200072, China; Zhejiang Engineering Research Center for Tissue Repair Materials, Wenzhou Institute, University of Chinese Academy of Sciences, Wenzhou 325000, China; Zhejiang Engineering Research Center for Tissue Repair Materials, Wenzhou Institute, University of Chinese Academy of Sciences, Wenzhou 325000, China; Orthopedic Institute, Department of Orthopaedic Surgery, The First Affiliated Hospital, School of Biology & Basic Medical Sciences, Suzhou Medical College, Soochow University, Suzhou, Jiangsu 215006, China; Shanghai Engineering Research Center of Tooth Restoration and Regeneration & Tongji Research Institute of Stomatology & Department of Prosthodontics, Shanghai Tongji Stomatological Hospital and Dental School, Tongji University, Shanghai 200072, China; Shanghai Engineering Research Center of Tooth Restoration and Regeneration & Tongji Research Institute of Stomatology & Department of Prosthodontics, Shanghai Tongji Stomatological Hospital and Dental School, Tongji University, Shanghai 200072, China

**Keywords:** copper hydrogen phosphate, diabetic periodontitis, antioxidant, osteogenic, antibacterial

## Abstract

Periodontitis, a chronic inflammatory disorder primarily induced by bacterial infection and exacerbated by excessive oxidative stress, leads to the destruction of alveolar bone. Diabetes mellitus intensifies this oxidative stress in periodontal tissues and disrupts the oral microbiome, thereby aggravating periodontal conditions and complicating the management of periodontitis. The development of materials that possess comprehensive therapeutic effects, including antibacterial, antioxidant and osteogenic properties, for the treatment of diabetic periodontitis (DP) remains at the forefront of research. In this study, we introduced a copper hydrogen phosphate (CuHP) composite hydrogel, which exhibited multi-enzymatic activities at varying pH levels. This hydrogel was synthesized by encapsulating CuHP within a commercially available sodium alginate (SA) matrix. *In vitro* analyses explored the pH-responsive enzymatic activities, biocompatibility and the antioxidant, osteogenic and antibacterial properties of the resultant SA/CuHP composite hydrogel. At neutral pH, the hydrogel primarily exhibited catalase-like activity, providing it with antioxidant capabilities that reduced the inhibitory effects of oxidative stress on osteogenesis in bone marrow mesenchymal stem cells. In mildly acidic conditions, the hydrogel displayed peroxidase-like activity, catalysing the production of more potent reactive oxygen species and exhibiting significant antibacterial efficacy against *Aggregatibacter actinomycetemcomitans*. Furthermore, the SA/CuHP hydrogel continuously released copper ions, which synergistically enhance its osteogenic and antimicrobial efficacies. *In vivo* studies demonstrated that this composite hydrogel significantly inhibited bacterial growth and promoted bone regeneration in a rat model of DP. These findings suggest that the SA/CuHP hydrogel holds substantial potential for the treatment of periodontitis in patients with diabetes.

## Graphical abstract

**Figure:**
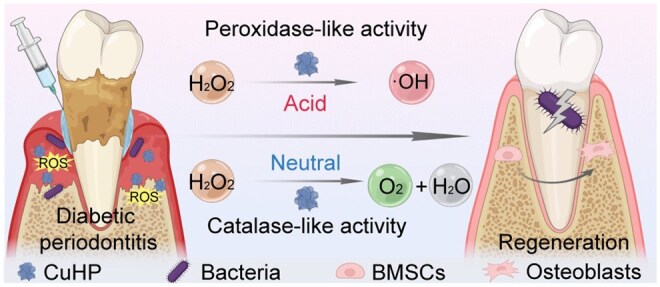

## Introduction

Periodontitis, a chronic inflammatory disorder affecting the periodontal tissues, is initially triggered by bacterial infection and further exacerbated by a dysregulated immune response from the host [1]. This pathological process leads to the progressive destruction of the supporting structures of the periodontium, notably the alveolar bone [2]. Moreover, diabetes mellitus significantly intensifies the progression of periodontitis through a complex interplay of factors [3]. High-glucose environment in the gingival fluid of diabetic individuals supports the proliferation of specific bacteria, thereby driving microbial dysbiosis that prompts the development of periodontitis [4]. In the progression of diabetic periodontitis (DP), bacteria not only cause direct damage to periodontal cells but also trigger excessive reactive oxygen species (ROS) generation from the host [5]. Concurrently, hyperglycaemia induces ROS overproduction while impairs antioxidant defences, synergistically redox imbalance in periodontal tissues [6]. This altered oxidative environment promotes cellular apoptosis and compromises osteoblast differentiation, culminating in accelerated resorption of the alveolar bone [7]. Consequently, bacterial infection, oxidative stress and impaired osteogenic activity each play unique and non-negligible pathogenic roles in the progression of DP. Recent clinical trials suggest that the adjunctive use of locally administered therapeutic agents possessing antibacterial, antioxidant and osteogenic properties, in conjunction with scaling and root planning, can significantly improve treatment outcomes [8–11]. Despite these advancements, the management of DP continues to pose a significant clinical challenge [12]. Therefore, it is imperative to develop new strategies that effectively target the eradication of pathogenic bacteria, neutralisation of excessive ROS and enhancement of osteogenesis to improve the efficacy of treatments for DP.

Recent investigations into catalytic biomaterials that exhibit inherent enzyme-mimetic properties have shown promise in the treatment of periodontitis, particularly those materials demonstrating oxidoreductase-like activities [13–15]. For example, biomaterials with oxidase (OXD)-like and peroxidase (POD)-like functionalities are capable of annihilating periodontal pathogenic bacteria and dismantling biofilms through the catalysis of reactive ROS generation, such as •OH [16, 17]. In contrast, materials that exhibit superoxide dismutase (SOD)-like and catalase (CAT)-like activities function analogously to antioxidant enzymes, scavenging ROS and thereby reducing the inhibitory effects of oxidative stress on osteogenesis [18–21]. However, research on the application of catalytic biomaterials for the treatment of DP remains sparse, with only a limited number of studies exploring this facet [22, 23]. A potential explanation for this gap is that the catalytic materials described to date often exhibit relatively singular functionalities, lacking the ability to independently address the combination of needs—namely, the reduction of excessive ROS for antioxidation, the promotion of osteogenesis and the generation of ROS for antibacterial action—required in DP [24, 25]. Consequently, there is a pressing need to develop multifunctional catalytic materials that possess antibacterial, antioxidant and osteogenic properties tailored for the management of DP.

Research has demonstrated that various copper-containing materials possess outstanding enzymatic properties [26–29]. In our prior studies, we developed a copper-based material that exhibited POD-like activity under mildly acidic conditions and CAT-like activity in neutral environments, which proved highly effective in therapeutic applications for wound infections and tracheal mucosal defects [30, 31]. Moreover, copper is critical for bone collagen growth and maturation and skeletal development, making it integral to osteogenesis [32]. Notably, the normal oral pH typically ranges within the neutral spectrum, whereas periodontal microenvironment become acidic during periodontitis [33, 34]. Although effective clearance of bacteria restores oral pH to neutral levels, high concentrations of ROS persist in the periodontium even after control of bacterial infection [35, 36]. These ROS continue to cause oxidative damage to periodontal tissues and drive the ongoing process of bone resorption [37]. Accordingly, the copper-based catalytic material (Cu_4_H(PO_4_)_3_·3H_2_O, CuHP) that we previously synthesized, which exhibits multiple enzyme-like activities at varying pH levels, holds significant potential to enhance the treatment efficacy for DP. In an acidic microenvironment, CuHP eliminates pathogenic bacteria by mimicking the activity of POD; in the neutral conditions after control of bacterial infection, CuHP may utilize its mimicked CAT activity to exert antioxidant effects and concurrently promotes osteogenic differentiation of periodontal tissues. Therefore, we hypothesize that materials based on CuHP could provide an integrated approach to antioxidant, osteogenic and antibacterial treatment in DP.

Among the various local drug delivery methods for periodontitis, injectable hydrogels are particularly notable due to their minimal invasiveness and their ability to achieve uniform distribution within the irregularly shaped periodontal pockets [38, 39]. Sodium alginate (SA), derived from natural sources, is abundantly available, highly biodegradable and readily crosslinked with divalent ions such as copper and calcium ions. These properties make SA an optimal candidate for the creation of injectable hydrogels that offer efficient and stable drug delivery capabilities [40].

**Figure 1.**
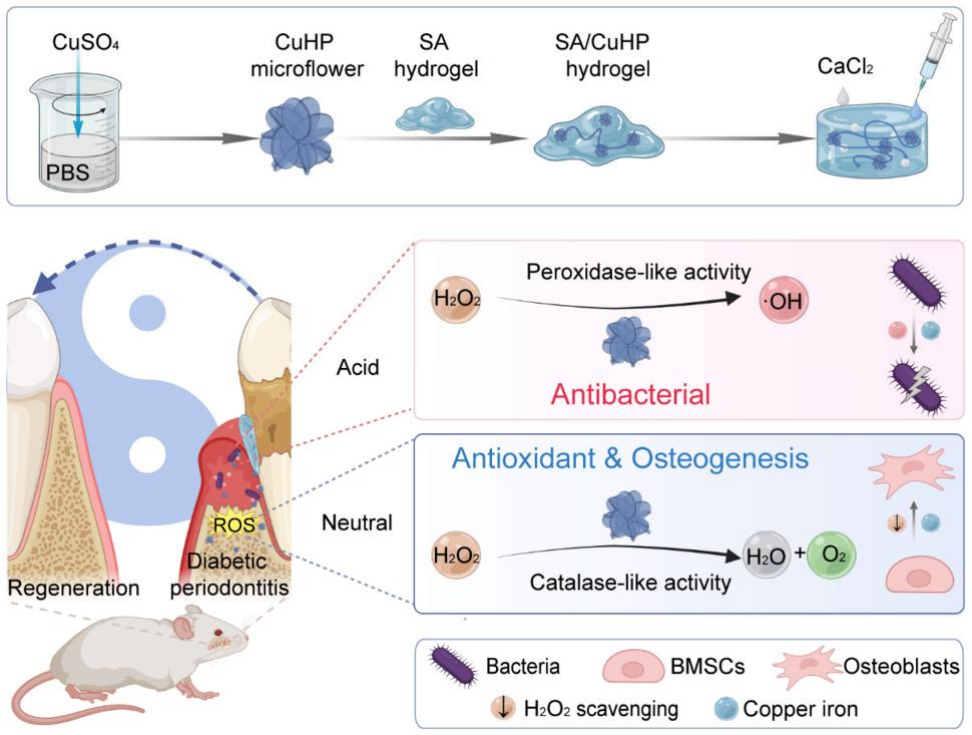
Schematic illustration depicting the preparation of the SA/CuHP hydrogel and its application in treating DP. This diagram highlights the antioxidant, osteogenic and antibacterial properties of the hydrogel, attributed to its pH-responsive multi-enzyme mimicking capabilities. The illustration was created using BioRender.com.

Building on this foundation, we have developed an innovative approach for treating DP. This involves the encapsulation of synthesized CuHP within an SA hydrogel (Figure 1). The resultant SA/CuHP hydrogel exhibited excellent injectability, multi-enzyme activities at various pH levels and favourable biocompatibility. We conducted *in vitro* studies to explore the antioxidant, osteogenic and antibacterial effects of this composite hydrogel. Remarkably, in a neutral environment, the hydrogel’s CAT-like activity provided it with superior intracellular antioxidant potential. The hydrogel notably enhanced the proliferation, viability, migration and osteogenic differentiation of bone marrow-derived mesenchymal stem cells (BMSCs) under conditions of oxidative stress. Additionally, in mildly acidic conditions, its POD-like activity imparted effective antibacterial properties. Furthermore, the SA/CuHP hydrogel facilitated the sustained release of copper ions, which inherently possessed osteogenic and antibacterial properties, thereby synergistically enhancing therapeutic efficacy. In a rat model of DP, the SA/CuHP hydrogels demonstrated significant antibacterial and osteogenic effectiveness. Overall, our research aims to introduce a novel therapeutic strategy for the management of DP.

## Materials and methods

### Materials

Copper sulphate (CuSO_4_) and Copper chloride (CuCl_2_) was procured from Macklin Co., Ltd. (Shanghai, China). Calcium chloride (CaCl_2_) was sourced from Aladdin Co., Ltd. (Shanghai, China). Alpha minimum essential medium, high-glucose Dulbecco’s modified Eagle’s medium (H-DMEM), phosphate-buffered saline (PBS) and penicillin/streptomycin (PS) were obtained from HyClone Co., Ltd. (Logan, UT, USA). Fetal bovine serum (FBS) was purchased from ExCell Bio Co., Ltd. (Shanghai, China).

### Preparation of CuHP and CuHP containing SA composite hydrogel (SA/CuHP)

The synthesis of CuHP and SA/CuHP hydrogel was conducted according to previously described methods [30]. Initially, 0.768 g of CuSO_4_ powder was dissolved in 10 mL of ultrapure water. The resultant solution was then mixed with 500 mL of 0.02 M PBS. The mixture was agitated and allowed to react for 6 h to ensure thorough mixing and complete reaction. Following this, the supernatant was removed by centrifugation at 10 000 rpm for 5 min. The CuHP were subsequently isolated and purified through three successive washes. To fabricate the SA/CuHP composite hydrogels, 1.5 g of SA was dissolved in 100 mL of double-distilled water to create a 1.5% (w/v) SA solution. CuHP were then incorporated into the SA solution at varying concentrations of 0.25%, 0.5%, 1% and 2% (w/v). Each mixture was crosslinked using a 1% (w/v) solution of CaCl_2_ to form SA/CuHP hydrogels at the designated concentrations: 0.25%, 0.5%, 1% and 2%. A control SA hydrogel was also prepared using a similar procedure but without adding CuHP. Additionally, to assess the biological implications of copper ion release, a CuCl_2_-loaded SA hydrogel (SA/CuCl_2_) was synthesized employing a method analogous to that used for SA/CuHP. The dosage of CuCl_2_ was adjusted to match the copper concentrations in the 1% SA/CuHP formulation.

### Characterization of CuHP and SA/CuP hydrogels

(i) To explore the morphologies of CuHP and SA/CuHP hydrogels, a Scanning Electron Microscope (SEM, model SU8010, HITACHI, Japan) was employed. (ii) The crystallographic structure of CuHP was analysed using X-ray Diffraction (XRD, model D8 ADVANCE, Bruker, Germany). (iii) Elemental mapping was performed with a Dimension ICON device (Bruker, USA). (iv) Mechanical characteristics of the hydrogels were evaluated using a mechanical tester (model 5982, INSTRON) following previously described methods [41]. Cylindrical samples of both SA and SA/CuHP hydrogels, measuring 10 mm in diameter and 5 mm in height, were subjected to uniaxial compression at a strain rate of 1 mm/min. The elastic modulus was calculated from the linear portion of the stress-strain curve within the 0–10% strain range, while the compressive strength was defined as the maximum stress observed prior to structural failure. (v) Rheological properties were assessed using a rheometer (model Mars40, Thermo Fisher, USA). The storage modulus (G’) and loss modulus (G”) were measured at a frequency of 1 Hz according to previously described methods [41]. (vi) Gelation properties and injectability of the hydrogels were assessed through visual inspection. (vii) To evaluate the adhesive properties of the hydrogels, porcine skin samples were prepared by cutting them into dimensions of 4 × 2.5 cm^2^. Subsequently, 100 µL of SA/CuHP hydrogel was applied to one side of each skin sample, covering an area of 2.5 × 1 cm^2^, according to previously reported methods [38]. The treated pieces were then bonded on the opposing sides. The adhesive strength of the hydrogels was quantitatively assessed by measuring the maximum weight that the bonded interface could sustain without failure. (viii) For the assessment of swelling ratio, SA and SA/CuHP hydrogels were synthesized and their initial masses (M0) were recorded. These samples were then immersed in a PBS solution maintained at 37°C. The mass of each hydrogel (Mn) was periodically recorded. The swelling ratio (%) was calculated using the formula: (Mn − M0)/M0 × 100% as previously described [42].

The POD-like performance of SA/CuHP hydrogels were evaluated according to previously reported methods [30]. First, various concentrations of the hydrogels (0.25%, 0.5%, 1% and 2%) were introduced into a 0.2 mL of 0.015% methylene blue (MB) solution. Then, the mixtures were agitated for 20 min before the addition of a 0.8 mL of 3% H_2_O_2_ solution. The reactions were allowed to proceed at ambient temperature (AT) for 10 min, following which the change in absorbance at 664 nm was quantified using UV–Visible–Near infrared (UV–Vis–NIR) spectrophotometry. To further explore the POD-like activity of SA/CuHP across different pH values (5, 6 and 7.4), a 1% SA/CuHP hydrogel was added to the 0.2 mL of 0.015% MB solution at these specified pH levels for 20 min. The CAT-like activity of the 1% SA/CuHP hydrogel across different pH values (5, 6 and 7.4) was assessed utilising a CAT assay kit (Visible light) (Nanjing Jiancheng, China), in accordance with the manufacturer’s instructions and the absorbance change at 402 nm was determined using a microplate reader (BioTek Synergy HIM, USA). Then, H_2_O_2_ scavenging rate was calculated according to previously described methods [43]. Additionally, the SA/CuHP hydrogel was dissolved in a 10% H_2_O_2_ solution adjusted to pH levels of 5, 6 and 7.4, wherein the evolution of O_2_ bubbles was observed.

### Copper ion release properties of SA/CuHP hydrogels

Copper ion release properties of SA/CuHP hydrogels were conducted following previously described methods [30, 41]. Initially, 0.02 g of SA/CuHP hydrogel was introduced into 20 mL of PBS. These samples were incubated at 37°C, and the supernatants were collected at 1, 3 and 5 days. Subsequently, inductively coupled plasma mass spectrometry (7850, Agilent, Singapore) was employed for quantifying the released copper ion level in these supernatants.

### Isolation and culture of BMSCs

Maxillary bone tissue was harvested from 4-week-old female Sprague–Dawley (SD) rats for the extraction of BMSCs following an established protocol [44]. The tissue was meticulously rinsed and dissected into fragments measuring 1-2 mm^2^. Subsequently, these fragments were grown in αMEM medium, containing 1% PS and 10% FBS, at a constant temperature of 37°C. We selected cells from passages 3–5 to carry out further experimentation. These processes employed a complete H-DMEM containing 1% PS and 10% FBS to provide a high-glucose condition.

### Biocompatibility *in vitro*

The biocompatibility of the SA/CuHP hydrogels was assessed *in vitro* using the CCK8 assay according to previous studies [30, 31]. The experiment involved seeding 2000 BMSCs into each well of a 96-well plate and letting them develop for one day. The cells were then co-cultured with various concentrations of SA/CuHP hydrogels (0.25%, 0.5%, 1% and 2%) or SA alone, using a volume of 10 µL, at 37°C for 5 days. On Days 1, 3 and 5, each well received 10 µL of CCK8 solution and was subsequently incubated at 37°C for 1 h. To assess cell viability, absorbance readings were taken at 450 nm.

### Antioxidant effect *in vitro*

Initially, conditions to establish an H_2_O_2_-induced oxidative stress model in a high-glucose environment were explored, based on previous studies [45–48]. To better simulate high-ROS microenvironment in DP, a dual-stimulus model was employed, combining H-DMEM medium (25 mM glucose) to simulate a high-glucose environment with H_2_O_2_ treatment to synergistically induce oxidative stress. First, 4000 BMSCs per well were incubated in a 96-well plate for a day. The cells were then exposed to either 300 μM or 500 μM H_2_O_2_ in complete H-DMEM for 3 h, followed by a recovery period in normal medium for a day. The establishment of the oxidative stress model was confirmed using a 500 μM H_2_O_2_ treatment for 3 h, as assessed by the CCK8 assay.

To evaluate the antioxidant capacity of the SA/CuHP hydrogel, further experiments were conducted. 4000 cells per well were placed in a 96-well plate and left to incubate for a day to standardize initial conditions across all groups. The experiments encompassed the Control group, the H_2_O_2_ group, the H_2_O_2_ + SA group and the H_2_O_2_ + SA/CuHP group. Treatments involved the application of either SA or SA/CuHP hydrogels, along with 500 μM H_2_O_2_ for 3 h. Cells in the Control group received no treatment, while those in the H_2_O_2_ group were exposed to 500 μM H_2_O_2_ without hydrogels. After the treatments and an additional 24-h incubation period, we employed the CCK8 assay for quantifying cell viability according to previous studies [21]. Additionally, to specifically ascertain the effects of copper ion release on cellular viability under oxidative stress, an experimental group treated with H_2_O_2_ + SA/CuCl_2_ was established, mirroring the protocol used for the H_2_O_2_ + SA/CuHP group but differing solely in the hydrogel composition.

Moreover, we undertook assays for further investigating the cytoprotective effects of the SA/CuHP hydrogel against oxidative stress, based on previous studies [30, 49]. These assays included: (i) 2,7-Dichlorofluorescein Diacetate (DCFH-DA) Staining Assay: BMSCs were plated at a density of 25 000 cells per well in a 24-well plate and cultured for a day. Following various treatments over a period of 3 h and a subsequent 24-h incubation period, as previously described, the cells were exposed to a DCFH-DA probe for 30 min. Fluorescence microscopy was employed for visualisation using a microscope (ZEISS, Germany). (ii) Live/Dead Cell Staining Assay: 50 000 cells per well were placed in a 12-well plate for a day. Post-treatment and incubation, as outlined above, the live and dead cells were stained using a Calcein-AM/PI Kit (Beyotime, China) and subsequently imaged with a microscope (Olympus, Japan). (iii) Cell Scratch Assay: Cells were seeded at a density of 100 000 cells per well in a 6-well plate and cultured for one day. A straight line was then scratched on the bottom surface of each well using a 200 μL pipette tip and cellular debris was removed with PBS. Following the treatment and incubation protocols mentioned earlier, cell migration was observed and documented at 0 and 24 h using a microscope.

### Osteogenic differentiation *in vitro*

To evaluate the impact of the SA/CuHP hydrogel on the osteogenic differentiation of BMSCs under oxidative stress conditions, we analysed the alkaline phosphatase (ALP) activity, calcium nodule formation and relative expression of osteogenic genes according to previous studies [21, 44, 50]. BMSCs were seeded at a density of 50 000 cells per well in a 12-well plate and cultured for a day. The experiments were structured into four groups, as previously mentioned. Following various treatments for 3 h, an osteogenic induction medium containing 10% FBS, 1% PS, 50 μg·mL^−1^ L-ascorbic acid (Sigma, USA), 100 nM dexamethasone (Sigma, USA) and 10 mM β-glycerophosphate (Sigma, USA) was administered over periods of 4, 7 or 21 days.

#### Assays for ALP and Alizarin red solution staining

On Day 7, an ALP staining kit (Beyotime, China) was employed for assessing ALP activity following the manufacturer’s instructions. Additionally, to elucidate the osteogenic effects mediated by copper ion release under oxidative stress, a group featuring H_2_O_2_ + SA/CuCl_2_ was established, maintaining identical treatment and osteogenic induction protocols as the H_2_O_2_ + SA/CuHP group, with the only variable being the hydrogel composition. The ALP activity for the H_2_O_2_ + SA/CuCl_2_ group was measured on Day 7. On Day 21, calcium nodule formation was assessed by staining the cells with Alizarin red solution (ARS) (OriCell, China) as per the provided guidelines. The stained cells and calcium nodules were observed using a microscope and a semi-quantitative analysis was conducted.

#### Immunofluorescence staining assay

On Day 4, samples were initially fixed in a 4% paraformaldehyde solution for 30 min, followed by three subsequent 5-min washes with PBS. The samples were then permeabilized using 0.25% Triton X-100 at AT for 5 min. After a blocking step involving 5% BSA (Beyotime, China) at AT for 1 h, the samples were incubated overnight at 4°C with a Runt-related transcription factor 2 (RUNX2) primary antibody (1:200, Proteintech, China). Following another series of three 5-min washes with PBS, the samples were incubated with a secondary antibody (1:200, Proteintech, China) at AT for 1 h in the dark. A drop of DAPI-containing anti-fluorescence quencher was then added to each dish. Immunofluorescence (IF) images were subsequently captured using a fluorescence microscope and analysed semi-quantitatively.

#### Quantitative reverse transcription polymerase chain reaction

On Day 4, total RNA was extracted from cells using Trizol reagent (TaKaRa, Japan). A Nanodrop spectrophotometer was employed for assessing the RNA concentration and purity. A PrimeScript RT reagent Kit (TaKaRa, Japan) was used for reverse transcribing the RNA into cDNA. A defined quantity of cDNA, along with forward and reverse primers (Supplementary Table S1), SYBR Green PCR Master Mix (Yeasen, Shanghai, China) and DEPC water, was added to a quantitative reverse transcription polymerase chain reaction (qRT-PCR) instrument (Roche, Switzerland) to facilitate the amplification process and obtain CT values. Relative gene expression levels were computed and normalized against GAPDH using the 2^−ΔΔCt^ method.

#### Western blot analysis

On Day 4, cells were lysed using RIPA buffer (Beyotime, China) at 4°C for 20 min. The lysate was centrifuged to collect the supernatant containing the protein samples. Protein concentrations were determined using a BCA assay kit (Beyotime, China) and adjusted to uniformity. Protein samples were then mixed with loading buffer and heated at 100°C for 5 min. Proteins were separated by SDS-PAGE and transferred onto 0.22 μm nitrocellulose membranes (Millipore, USA) using a wet transfer method. After blocking with 5% nonfat milk (Elabscience, China) at AT for 1 h, the membranes were incubated overnight at 4°C with primary antibodies against GAPDH (1:1000, Cell Signaling, USA), RUNX2 (1:1000, Abcam, UK) and OSX (1:1000, Abcam, UK). Following washes with TBST buffer, the membranes were incubated with a secondary antibody (1:1000, Proteintech, China) at AT for 1 hr. After additional washes, enhanced chemiluminescence reagent was applied and the proteins were visualized using a chemiluminescence imager (Tanon 4600, Shanghai, China). Protein expression levels were analysed based on band position and intensity.

### RNA sequencing

To elucidate the osteogenic mechanisms of the SA/CuHP hydrogel under oxidative stress conditions, transcriptomic analyses were undertaken using a method previously described [51]. Initially, BMSCs were exposed to either H_2_O_2_ alone (H_2_O_2_ group) or H_2_O_2_ combined with SA/CuHP (SA/CuHP group). These treatments were facilitated using Trizol reagent (Takara, Japan). Subsequent steps involved RNA extraction and sequencing, which were meticulously carried out by Majorbio Bio-pharm Technology Co., Ltd. (Shanghai, China). Differential gene expression was determined using thresholds of *P* < 0.05 and |log 2 Fold Change| ≥ 1. Gene Ontology (GO) enrichment analysis and Kyoto Encyclopedia of Genes and Genomes (KEGG) pathway analyses were conducted using the Majorbio Cloud Platform (www.majorbio.com).

### Antibacterial activity *in vitro*

Antibacterial assessments were performed utilising plate counting, live/dead staining and SEM observation, based on reported studies [22, 52]. *Aggregatibacter actinomycetemcomitans* (*A.a*, ATCC 33384, G-) served as the bacterial model in these *in vitro* experiments. The bacteria were cultured in anaerobic basal broth (QDRS Biotech, China) at 37°C. The experimental design included six groups: the Control, the H_2_O_2_, the SA, the H_2_O_2_ + SA, the SA/CuHP and the H_2_O_2_ + CuHP/SA. Each bacterial solution was initially diluted to 10^6^ CFU mL^−1^. Subsequently, 50 μL of various hydrogels (either SA or SA/CuHP) and 500 μM H_2_O_2_ (±) were used for culturing 1 mL of the solution in anaerobic basal broth (pH = 5, 37°C, 24 h). The untreated bacteria served as the Control group. The evaluation of antibacterial activity included: (i) stepwise serial dilution of the bacterial suspension from 10^−5^–10^−7^, followed by overnight culture on anaerobic agar plates (Hopebio, China) at 37°C, with subsequent recording of colony-forming unit counts; (ii) assessment of bacterial viability using the Live/Dead BacLight Bacterial Viability Kit (Thermo Scientific, USA), adhering to the manufacturer’s instructions; and (iii) observation of bacterial morphology via SEM. Post-treatment, the bacteria were washed with PBS, fixed in 2.5% glutaraldehyde at 4°C for 6 h, dehydrated using a graded ethanol series, dried and gold-sputtered for SEM observation.

### Establishment of a periodontitis model in type 2 diabetic rats

This study received ethical approval from the Animal Welfare Committee of Tongji University Stomatological Hospital (2020-DW-02). The protocol for establishing a type 2 DP model in rats was adapted from previously described methods [30, 49, 53]. Male Sprague–Dawley rats, aged 4 weeks and weighing between 70 and 100 g, were subjected to a high-fat diet containing 60% fat (Research Diets, SYHF60-1) for 4 weeks. Following this, the rats were administered an intraperitoneal injection of STZ, dissolved in sodium citrate buffer (pH 4.5, at a dosage of 35 mg/kg). Blood glucose levels (BGLs) were monitored on Days 3 and 7 using a glucose meter (Yuwell, China) and rats exhibiting random BGLs exceeding 16.7 mmol/l after 7 days were categorized as diabetic. The diabetic rats were subsequently randomized into three experimental groups (*n* = 6 per group): the PBS group, the SA group and the SA/CuHP group. Rats that did not receive any treatment constituted the Healthy group. Periodontitis was induced in the diabetic rats through silk ligature according to a described method [44]. The rats were anaesthetized with 10% chloral hydrate (30 mg/kg) via intraperitoneal injection and 3-0 silk sutures were subgingivally placed around the left maxillary second molar (M2) for 3 weeks. Following the removal of the ligature, 10 μL each of PBS, SA and SA/CuHP were injected into both the palatal and buccal periodontal pockets of M2 twice weekly.

### Retention and degradation of hydrogels *in vivo*

The retention of hydrogels was evaluated in the context of periodontitis in diabetic rats, induced by silk ligature as previously outlined. After the removal of the ligature, 10 μL of SA/CuHP hydrogel was administered into the periodontal pockets. The maxillae were harvested at intervals of 1 h, 1 day, 2 days and 3 days post-administration to assess the retention of the hydrogel. To enhance the visibility of the blue hydrogel, transcardial perfusion with PBS was performed to clear blood prior to euthanising the rats.

To assess the *in vivo* degradation of hydrogels, the rats were anaesthetized using methods previously described [41, 54]. Subsequently, 0.3 g of either SA or SA/CuHP hydrogels were implanted subcutaneously in the dorsal area. The hydrogels were harvested, photographed and weighed on Days 3 and 7 post-implantation. For baseline comparison, an equivalent amount of hydrogels was photographed on Day 0 as the Control group.

### Therapeutic effects of hydrogels on diabetic rats with periodontitis

Following a 2-week treatment period, the periodontal pockets of the M2 were swabbed using aseptic swabs, which were then saturated in sterile PBS to prepare a bacterial suspension using methods previously described [55]. This suspension was subsequently diluted and cultured on anaerobic agar plates at 37°C for a day. The resultant bacterial colonies were digitally photographed and quantified, providing a measure of the hydrogels’ antibacterial activity. After 4 weeks of treatment, the subjects were euthanized and their maxillae along with major organs were harvested for further analysis. The maxillary bones were fixed in 4% paraformaldehyde for 2 days. Scanning was performed using a Scanco mCT 50 (Scanco Medical, Switzerland) and 3D reconstruction images were generated following methods previously reported [44]. The linear distance from the cement-to-enamel junction to the alveolar bone crest (CEJ-ABC) was measured at various points including the mesial, central and distal aspects on both buccal and palatal surfaces of the M2 and mean values were computed. Additionally, sectional and radiographic images were obtained from buccal-palatal perspectives. For a detailed quantitative analysis, the interalveolar septum between the first and second molars was selected, focusing on parameters such as bone volume per tissue volume (BV/TV) and trabecular separation (Tb, Sp).

Further histopathological analyses were performed according to previously established protocols [44]. Post micro-CT analysis, the maxillae were decalcified in a 10% solution of EDTA (Greagent, Shanghai, China) over an 8-week period. Following decalcification, the samples underwent dehydration in an automatic dehydrator (Thermo Scientific, USA) and were embedded in paraffin wax (Leica, Germany) to ensure proper fixation. Subsequently, the samples were sectioned into 4-μm slices. Prior to staining, these sections were subjected to deparaffinisation and rehydration processes. Staining was then performed using Hematoxylin and Eosin (HE) (Beyotime, Shanghai, China) and a Masson staining kit (Keygen Biotech, Shanghai, China), in accordance with established protocols. The morphological features of the samples were examined under a microscope. Furthermore, to evaluate the systemic safety of the hydrogel treatment, significant organs including the spleen, heart, lung, kidney and liver were extracted from the rats, stained with HE and examined using a light microscope.

### Statistical analysis

Data were shown as mean ± standard deviation (SD). Images were first processed using ImageJ software to extract relevant quantitative data. GraphPad Prism 9 and Origin 2024 were used for further statistical analysis. One-way ANOVA was performed to assess the significant differences among different groups. Statistical significance was indicated as: ns, not significant, *P < 0.05, **P < 0.01, ***P < 0.001.

## Results and discussion

### Preparation and characterization of CuHP and SA/CuHP hydrogel

**Figure 2.**
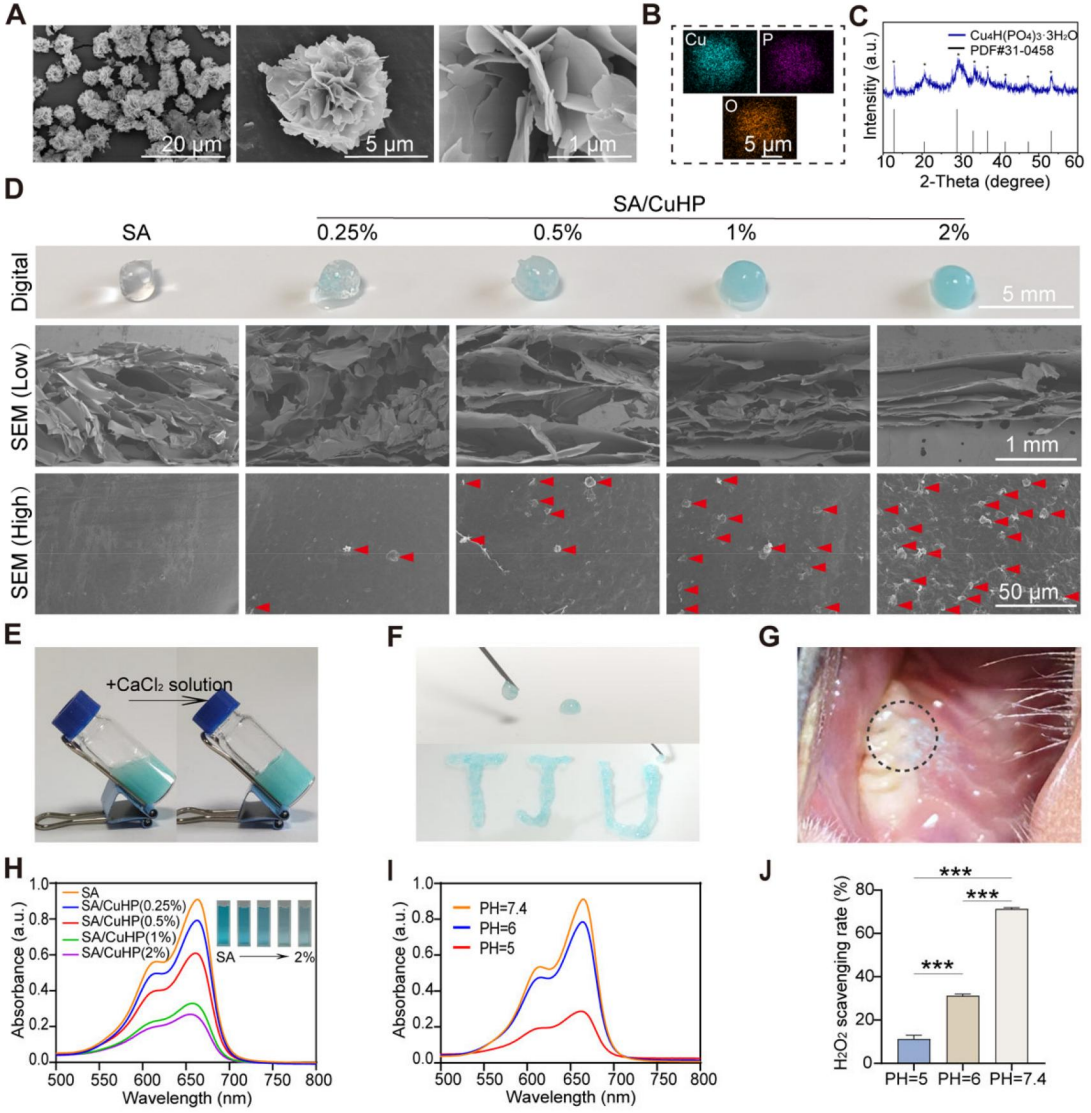
Characterisation of CuHP and SA/CuHP hydrogels. (**A**) SEM images of CuHP. (**B**) Element mapping for Cu, P and O in CuHP. (**C**) Characteristic XRD peaks of CuHP. (**D**) Digital and SEM images of SA/CuHP hydrogels at varying concentrations, with red arrows indicating the presence of CuHP within the hydrogels. (**E**) Observation of the injectable properties, (**F**) gelation and (**G**) *in situ* application of SA/CuHP hydrogels. (**H**, **I**) POD-like activity: degradation of methylene blue by SA/CuHP hydrogels at different concentrations and pH levels. (**J**) H_2_O_2_ scavenging rate of SA/CuHP hydrogel at 1% concentration and different pH levels. Data are presented as mean ± SD (*n* = 3).

The SEM images, as depicted in Figure 2A, illustrated that the synthesized particles exhibited a microflower-like structure comprised numerous flake-shaped nanosheets. This morphology provided a considerable specific surface area, thus, facilitating interactions with surrounding entities and enhancing catalytic reactions [56, 57]. Elemental mapping demonstrated a uniform dispersion of Cu, P and O elements, suggesting a homogeneous distribution of these elements (Figure 2B). The XRD pattern confirmed that the X-ray diffraction peaks of the sample precisely matched those of Cu_4_H(PO_4_)_3_· 3H_2_O (PDF No. 31—0458), providing essential insights into its crystalline structure (Figure 2C). These findings corroborate the successful synthesis of CuHP.

### Preparation and characterization of SA/CuHP hydrogel

Various concentrations of CuHP (0.25%, 0.5%, 1% and 2%, w/v) were incorporated into the SA hydrogel. The digital images revealed that the composite hydrogels exhibited an increasingly deep blue colour with the rising incorporation of CuHP, whilst the SA hydrogel remained colourless (Figure 2D). The SEM images indicated that the SA hydrogel possessed a porous structure, conducive to nutrient transmission and cell migration [55]. The CuHP was evenly and stably distributed throughout the hydrogel’s matrix without disrupting its porous framework. The compressive strength of the hydrogels was assessed (Supplementary Figure S1A–C). The SA/CuHP hydrogel demonstrated enhanced maximum stress and elastic modulus compared to the SA hydrogel alone. Rheological analysis revealed the viscoelastic properties of the hydrogels (Supplementary Figure S1D). The SA hydrogel displayed a condition where loss modulus (G”) exceeded storage modulus (G’), indicative of a sol state. Conversely, the SA/CuHP hydrogel exhibited G’ surpassing G”, suggesting that the incorporation of CuHP facilitated the sol-to-gel transition of the hydrogel. Upon interaction with the CaCl_2_ solution, SA/CuHP hydrogels transitioned from a sol to a gel state (Figure 2E). The sol-gel transition in the SA/CuHP hydrogel is derived from SA’s capability to form crosslinks with divalent cations such as copper and calcium ions [40, 42]. Furthermore, the injectability of the hydrogel was evaluated. Figure 2F illustrated that the SA/CuHP hydrogel possessed excellent injectability, easily forming various shapes. Figure 2G displayed the *in situ* application of the SA/CuHP hydrogel. Both SA and SA/CuHP hydrogels swelled rapidly at first and reached stabilisation within 24 h, with low swelling ratios (<100%) (Supplementary Figure S1E). As depicted in Supplementary Figure S1F, the adhesive interface (2.5 × 1 cm^2^) of SA/CuHP hydrogel afforded a shearing force of only about 0.05 N, indicative of its poor adhesion to tissue, consistent with previous researches indicating that commercial SA hydrogels typically exhibit poor adhesive properties [42, 58].

The enzymatic properties of SA/CuHP hydrogels, exhibiting both POD- and CAT-like activities, were further analysed. As illustrated in Figure 2H, a decrease in absorption at 664 nm and a corresponding lightening of the blue solution were observed with increasing concentrations of SA/CuHP hydrogels, indicating a positive correlation between the concentration of SA/CuHP hydrogels and their POD-like activity. Subsequently, the SA/CuHP hydrogel at a concentration of 1% was employed to evaluate the POD- and CAT-like activities across various pH values (pH 5, 6, 7.4). Optimal POD-like activity was recorded at pH 5 (Figure 2I), which was advantageous for its efficacy in the mildly acidic microenvironment typical of bacterial infections. As shown in Figure 2J and Supplementary Figure S2, the highest CAT-like activity of SA/CuHP hydrogel was observed at pH 7.4, as evidenced by the most pronounced O_2_ bubble generation and the highest H_2_O_2_ scavenging rate (71.3%), which was comparable to CAT-like materials reported in prior studies [21, 43].

### Copper ion release properties

Copper ions have been recognized as vital trace elements in human physiology, demonstrating multiple biological functions including angiogenesis promotion and osteogenic enhancement [59]. However, excessive copper ion concentrations (exceeding 10 μM) may induce cytotoxicity in BMSCs, as evidenced by prior studies [60]. To ensure biosafety, the copper ion release properties of SA/CuHP hydrogels were evaluated. As shown in Supplementary Figure S3, the composite hydrogels exhibited an initial 24-h burst-release phase followed by a sustained release over 7 days. Crucially, cumulative copper release positively correlated with CuHP loading amount. SA/CuHP hydrogels (0.25–1%) maintained cumulative copper ions release below the safety threshold, indicating biocompatibility within this range. Notably, the 2% SA/CuHP hydrogel exceeded critical levels, suggesting potential cytotoxic risk.

### Biocompatibility *in vitro*

**Figure 3.**
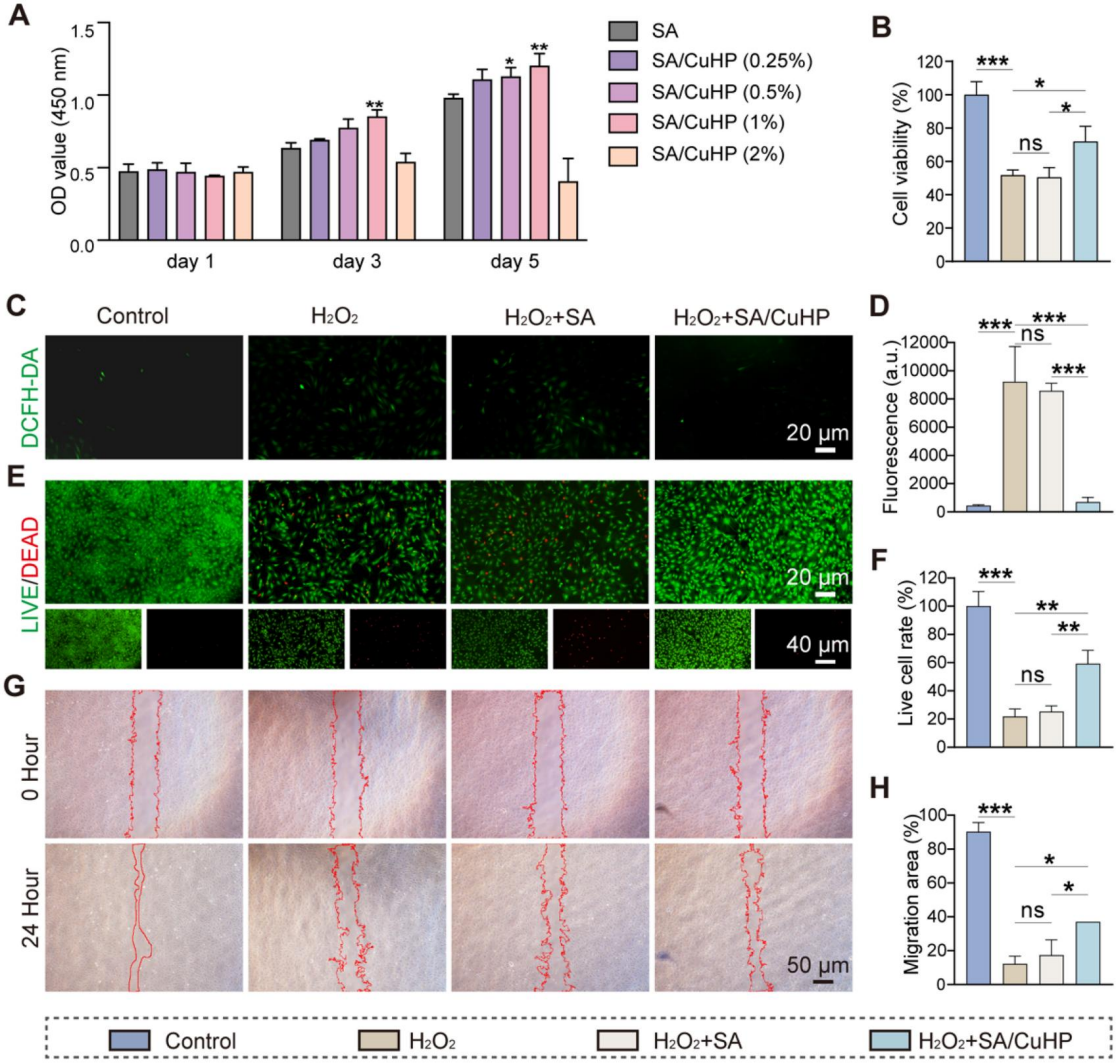
Biocompatibility and intracellular antioxidant ability of SA/CuHP hydrogel *in vitro*. (**A**) CCK8 cell proliferation assay conducted over 1, 3 and 5 days. (**B**) CCK8 cell viability assay conducted at 24 h. (**C**, **D**) Representative images (RIs) depicting DCFH-DA fluorescence along with semi-quantitative analyses of intracellular ROS levels at 24 h. (**E**, **F**) RIs of live/dead cell staining at 24 h, accompanied by semi-quantitative assessments of live cell percentages relative to the control group. (**G**, **H**) RIs from the cell migration assay and quantitative analyses conducted at 0 and 24 h. Control: BMSCs with no treatment; H_2_O_2_: BMSCs exposed to 500 μM H_2_O_2_ for 3 h; H_2_O_2_ + SA and H_2_O_2_ + SA/CuHP: BMSCs treated with SA or SA/CuHP hydrogels, respectively, along with 500 μM H_2_O_2_ for 3 h. Data are presented as mean ± SD (*n* = 3).

The assessment of cytotoxicity is imperative prior to the clinical application of biomedical materials [61]. To ascertain the optimal concentration of SA/CuHP hydrogels for further studies, BMSCs were cultured in the presence of various concentrations of these hydrogels and cellular proliferation was evaluated using the CCK8. The assay results on Days 1, 3 and 5 indicated that lower concentrations of SA/CuHP hydrogels (0.25% to 1%) enhanced cell proliferation, whereas a higher concentration (2%) exhibited cytotoxic properties. These findings were consistent with the characteristics of copper ion release. Notably, the SA/CuHP hydrogel at 1% concentration demonstrated significantly higher absorbance at 450 nm, indicative of the most pronounced promotion of BMSC proliferation within this group (Figure 3A). Consequently, the SA/CuHP hydrogel at this concentration was selected for subsequent experiments and will henceforth be referred to simply as SA/CuHP for ease of reference.

### Intracellular antioxidant ability *in vitro*

Hyperglycaemia fosters the production and accumulation of ROS, such as H_2_O_2_, through various mechanisms [62]. This increase in oxidative stress can impair cellular integrity and exacerbate bone-related disorders in periodontitis [63]. Consequently, the mitigation of excessive ROS is crucial for preserving cellular functionality and managing periodontitis in diabetic conditions [64]. To better mimic the high-ROS microenvironment of DP, cells were treated with a combination of H-DMEM medium (25 mM glucose) and H_2_O_2_ to synergistically induce oxidative stress [45–48]. Drawing on the results from the CCK8 cell viability assays, an *in vitro* oxidative model was established by subjecting BMSCs to 500 μM H_2_O_2_ in an H-DMEM culture medium for 3 h (Supplementary Figure S4).

As illustrated in Figure 3B and Supplementary Figure S5, cellular viability was significantly compromised following exposure to H_2_O_2_. In comparison with the H_2_O_2_-treated group, treatment with SA exhibited no restorative effects, whereas the application of the SA/CuHP hydrogel resulted in a significant enhancement in cell viability, affirming that SA/CuHP mitigated the cytotoxic effects induced by H_2_O_2_-driven oxidative stress. Low-dose copper ions have been proved to enhance cellular activity in previous studies [65]. To further investigate the effect of copper ion release, H_2_O_2_ + SA/CuCl_2_ group was conducted. Compared to H_2_O_2_ group, the H_2_O_2_ + SA/CuCl_2_ group only induced a marginal increase in cell viability, which was not statistically significant, suggesting insufficient antioxidant efficacy of copper ions and their suppressed capacity to promote cell viability under oxidative stress conditions.

To further assess the protective effects of the SA/CuHP hydrogel against oxidative stress, we employed DCFH-DA staining, live/dead staining and cell scratch assays. The production of intracellular ROS was confirmed via DCFH-DA staining, with pronounced green fluorescence observed in the H_2_O_2_ and H_2_O_2_ + SA groups, indicating elevated ROS levels compared to the Control group. Conversely, treatment with the SA/CuHP hydrogel effectively attenuated intracellular ROS levels (Figure 3C). The semi-quantitative analysis corroborated the trends observed in the DCFH-DA staining (Figure 3D), demonstrating the inhibitory effect of the SA/CuHP hydrogel on intracellular oxidation. As depicted in Figure 3E, the Control group exhibited a predominance of green-stained live cells. In contrast, increased cell death, indicated by red fluorescence, was evident in the H_2_O_2_ and H_2_O_2_ + SA groups. Remarkably, this cell death was effectively inhibited by treatment with the SA/CuHP hydrogel. The semi-quantitative results were consistent with the live/dead cell staining patterns (Figure 3F). Additionally, the cell scratch assay was utilized to evaluate cellular migration. As shown in Figure 3G and H, the migration rate of BMSCs in the H_2_O_2_ + SA/CuHP group was enhanced relative to the H_2_O_2_ and H_2_O_2_ + SA groups. These findings support the conclusion that SA/CuHP hydrogel effectively shields cells from oxidative stress-induced damage by eliminating ROS.

In summary, neither SA nor copper ions alone exhibited notable antioxidant properties. In stark contrast, the SA/CuHP hydrogel demonstrated remarkable antioxidative and cell-protective effects under oxidative conditions. These effects are likely attributable to the CuHP component, which predominantly exhibits CAT-like activity in a neutral environment. Previous studies have established that CAT-like biomaterials catalyse the decomposition of H_2_O_2_ into H_2_O and O_2_ [66]. For instance, Xie *et al*. developed a cobalt oxide-supported iridium (CoO–Ir) that functioned as a SOD- and CAT-like cascade artificial antioxidase, providing significant cellular protection against ROS damage [21]. Thus, it is hypothesized that the CAT-like activity of CuHP enhances the antioxidative properties by reducing the H_2_O_2_ in the environment and reducing the overproduction of intracellular ROS, thereby further protecting cells from the exacerbating effects of oxidative stress.

### Osteogenic response under oxidative stress *in vitro*

**Figure 4.**
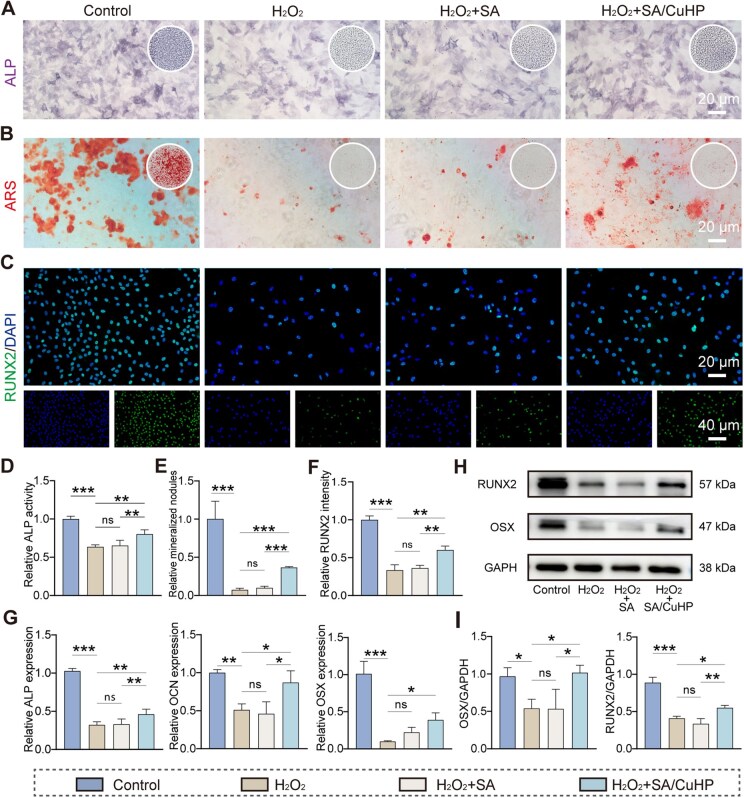
Osteogenic effects of SA/CuHP hydrogel under oxidative stress *in vitro*. (**A**) RIs of ALP staining on Day 7. (**B**) RIs of ARS staining on Day 21. (**C**) RIs of RUNX2 IF staining on Day 4. (**D**) Semi-quantitative results of ALP staining. (**E**) Semi-quantitative results of ARS staining. (**F**) Semi-quantitative results of RUNX2 IF staining. (**G**) Osteogenic-related gene expression of ALP, OCN and OSX measured by qRT-PCR on Day 4. (**H**, **I**) Protein expression of RUNX2 and OSX measured by Western blot and semi-quantitative results on Day 7. Control: BMSCs without treatment; H_2_O_2_: BMSCs exposed to 500 μM H_2_O_2_ for 3 h; H_2_O_2_ + SA and H_2_O_2_ + SA/CuHP: BMSCs treated with SA or SA/CuHP hydrogels, respectively, along with 500 μM H_2_O_2_ for 3 h. Data are presented as mean ± SD (*n* = 3).

BMSCs play a pivotal role in the regeneration of periodontal tissues [67]. They facilitate the differentiation of cells into osteoblasts, thereby promoting the formation of alveolar bone [68]. Nevertheless, in diabetic conditions characterized by elevated ROS, a significant inhibition of osteogenic differentiation in BMSCs occurs, exacerbating the destruction of alveolar bone [64]. As depicted in Figure 4A, the BMSCs treated with H_2_O_2_ and SA/CuHP hydrogel exhibited markedly enhanced ALP expression compared to those treated with H_2_O_2_ alone or H_2_O_2_ and SA. Notably, the group treated with H_2_O_2_ and SA/CuCl_2_ hydrogel displayed increased ALP activity when compared to the H_2_O_2_ group alone, although the osteogenic efficacy of the SA/CuCl_2_ hydrogel was still less than that of the SA/CuHP hydrogel, as shown in Supplementary Figure S6. This suggested that the release of copper ions played a role in enhancing osteogenic potential under oxidative conditions. ARS staining highlighted the ability of SA/CuHP hydrogel to promote osteoblast maturation, as evidenced by the formation of denser mineralized nodules compared with other groups exposed to H_2_O_2_ (Figure 4B). Additionally, the expression of RUNX2, a critical osteogenic marker in BMSCs, was assessed through IF staining. The H_2_O_2_ and H_2_O_2_ + SA groups exhibited reduced RUNX2 expression, whereas a significant increase in expression was observed in the H_2_O_2_ + SA/CuHP group (Figure 4C). Semi-quantitative analysis of ALP, ARS and RUNX2 IF staining corroborated the observed trends in staining (Figure 4D–F). Furthermore, the expression levels of osteogenic genes, including ALP, osteocalcin (OCN) and osterix (OSX), were analysed in BMSCs under oxidative stress using qRT-PCR. A marked downregulation of these genes was noted in cells exposed to H_2_O_2_, in contrast to their upregulation in the H_2_O_2_ + SA/CuHP group (Figure 4G). Notably, the SA/CuHP hydrogel restored the mRNA expression of OCN to 87% of the Control group level, a 1.7-fold increase relative to the H_2_O_2_ group (51% of the control level), demonstrating restoration efficacy of OCN expression comparable to the antioxidant-incorporated material reported in a prior study under oxidative stress [69]. The WB assays supported these findings, showing a reversal in the decline of RUNX2 and OSX protein expression induced by H_2_O_2_ following treatment with SA/CuHP hydrogel (Figure 4H and I).

Collectively, these results demonstrated the considerable capacity of SA/CuHP hydrogel to enhance the osteogenic potential of BMSCs under oxidative conditions. SA alone showed a modest osteogenic-promoting effect, consistent with findings from other studies [70, 71]. Treatment with SA/CuCl_2_ hydrogel increased ALP activity, suggesting that the release of copper ions contributes partially to the enhancement of osteogenesis under oxidative stress. Consequently, the osteo-protective efficacy of SA/CuHP hydrogel under such conditions can be attributed to the synergistic interaction between its antioxidant properties and the release of copper ions. Primarily, SA/CuHP hydrogel exhibited CAT-like enzyme activity in neutral conditions, which catalysed H_2_O_2_ into nontoxic water and oxygen under oxidative stress conditions. This characteristic helps mitigate the detrimental effects of excessive ROS on BMSCs. Previous research has reported similar properties in materials that scavenge ROS. For instance, Zhang *et al*. developed a factor-free hydrogel containing ROS-cleavable thioketal (TK) linkers, which not only scavenged ROS but also created a conducive microenvironment, thereby promoting osteogenic differentiation of BMSCs [49]. Additionally, the SA/CuHP hydrogel demonstrated a sustained release of bioactive copper ions, which synergistically enhance osteogenesis. Low doses of copper ions have been shown to exert a positive effect on the osteogenic process, such as upregulating osteogenic gene expression and stimulating bone matrix mineralisation [59]. Extensive research has supported the notion that copper-incorporated biomaterials significantly promote bone regeneration [72, 73].

**Figure 5.**
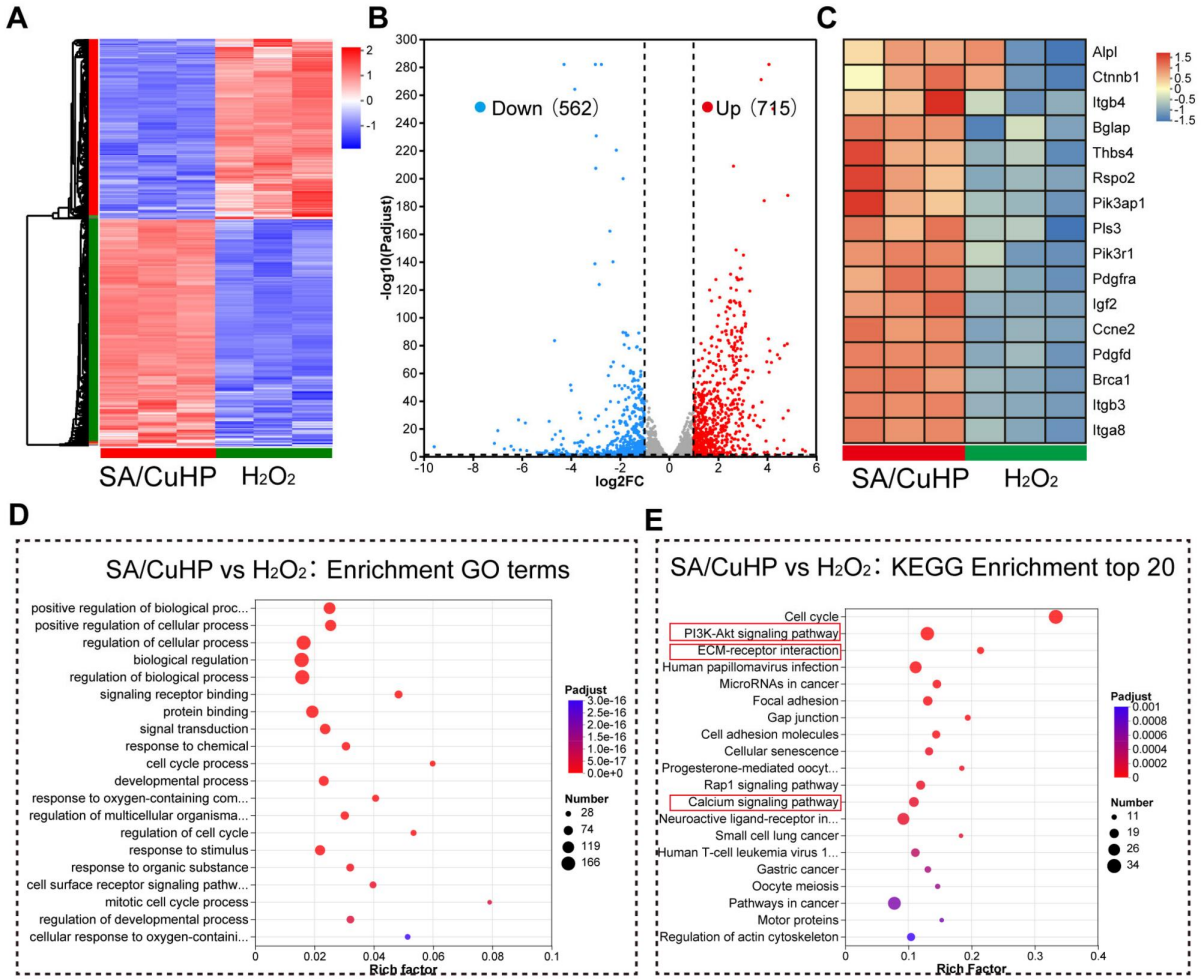
RNA-seq analysis of BMSC subjected to treatment with either H_2_O_2_ alone (H_2_O_2_ group) or a combination of H_2_O_2_ and SA/CuHP hydrogel (SA/CuHP group). (**A**) Heatmap depicting differentially expressed genes (DEGs) between treatment groups. (**B**) Volcano plot visualizing DEGs (threshold: *P* values < 0.05, |log 2 Fold Change| > 1). (**C**) Heatmap illustrating expression patterns of osteogenesis-associated genes. (**D**) Gene ontology (GO) enrichment analysis. (**E**) Top 20 signalling pathways via KEGG pathway analysis (*n* = 3).

RNA sequencing (RNA-Seq) is an indispensable tool for elucidating the genomic architecture and its functionality, as well as for identifying alterations in associated signalling pathways [74]. As represented in Figure 5A, the differential expression of genes in BMSCs treated with H_2_O_2_ (H_2_O_2_ group) or H_2_O_2_ + SA/CuHP hydrogel (SA/CuHP group) was characterized by red (upregulated genes) and blue (downregulated genes). The analysis of differentially expressed genes (DEGs) revealed 715 significantly upregulated and 562 downregulated genes (*P* < 0.05, |log2 Fold Change| > 1) in the SA/CuHP group compared to the H_2_O_2_ group (Figure 5B). Notably, a comparative analysis of genes exhibiting differential expression demonstrated that genes associated with osteogenesis, such as Alpl, Bglap, Ctnnb1, Igf2 and Rspo2, were upregulated in the SA/CuHP group relative to the H_2_O_2_ group (Figure 5C). To delineate the functional relevance of these DEGs, a GO enrichment analysis was conducted. As depicted in Figure 5D, intervention with SA/CuHP hydrogel profoundly influenced the regulation of cellular processes, biological regulation and response to stimuli, which are linked to oxidative stress adaptation and osteogenesis. Subsequently, a KEGG pathway enrichment analysis was performed to characterize the potential involvement of these DEGs in osteogenic signalling pathways (Figure 5E). Remarkably, these genes showed significant enrichment in pivotal antioxidant and bone formation-related pathways, including the “PI3K-AKT signalling pathway,” “ECM-receptor interaction” and “Calcium signalling pathway.” Notably, activation of the PI3K-AKT signalling pathway plays a pivotal role in promoting the efficient clearance of intracellular ROS [75, 76]. Furthermore, the activation of ECM-receptor interaction and the calcium signalling pathway has been shown to significantly enhance osteogenic differentiation in bone tissue regeneration [77, 78]. Based on these findings, we hypothesize that the SA/CuHP hydrogel exerts its beneficial effects by harnessing the antioxidant properties of the PI3K-AKT signalling pathway to mitigate ROS-induced damage, while simultaneously promoting osteogenic differentiation through the synergistic activation of ECM-receptor interaction and the calcium signalling pathway.

### Antibacterial efficacy *in vitro*

**Figure 6.**
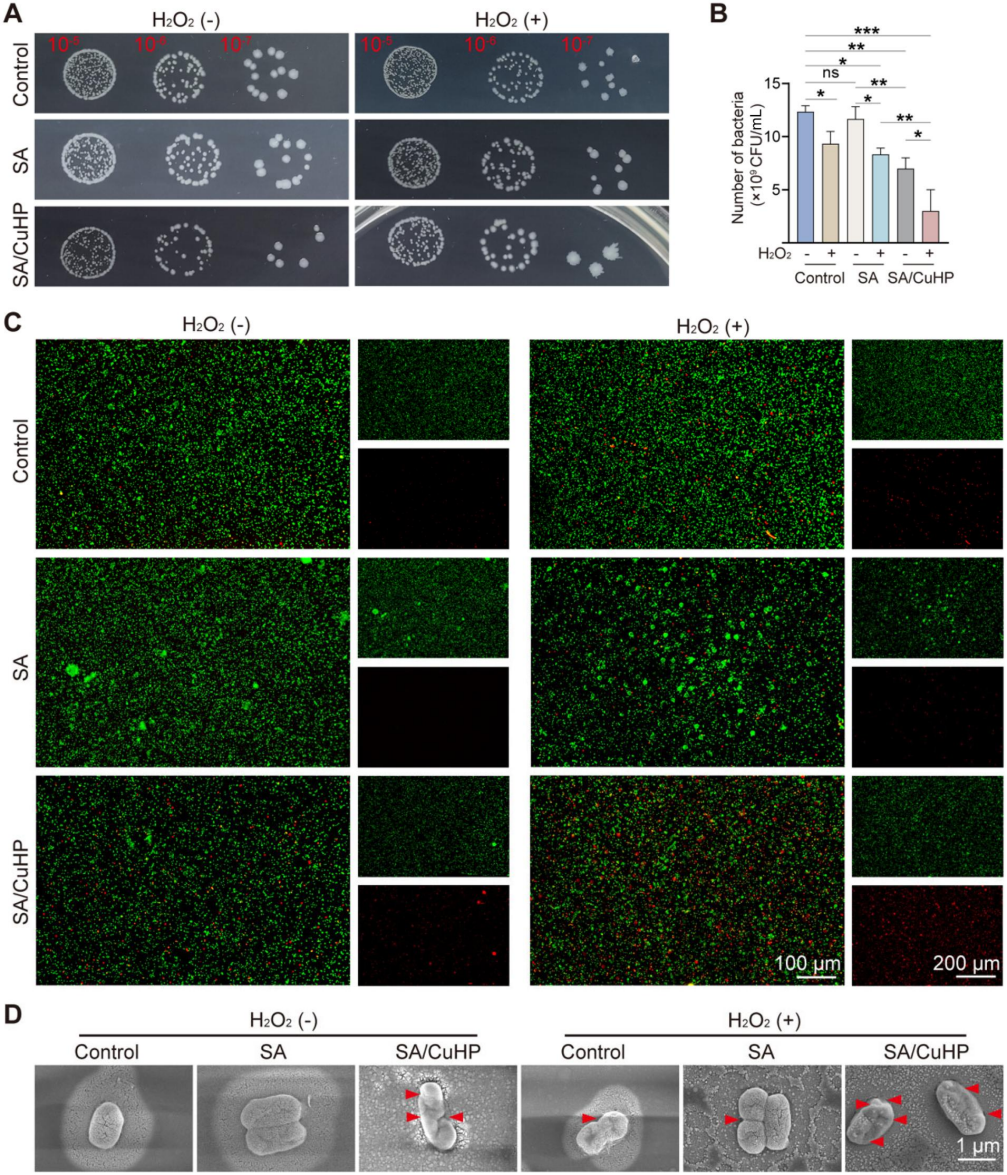
Antibacterial effect of SA/CuHP *in vitro*. (**A**) and (**B**) RIs of *A.a* bacterial colonies formed on agar plates and statistical analysis of colony counts. (**C**) RIs of *A.a* live/dead staining. (**D**) RIs of *A.a* SEM images, with red arrows indicating areas of membrane disruption. Data are presented as mean ± SD (*n* = 3).

Periodontitis is primarily caused by gram-negative bacteria, among which *A.a* plays a crucial role in the development of periodontitis [52, 79]. As demonstrated in Figure 6A and B, the colony formation on agar plates in both the SA and Control groups was comparable, suggesting that SA alone lacked significant antibacterial properties. A modest antibacterial action was observed in the H_2_O_2_, H_2_O_2_ + SA and SA/CuHP groups, with inhibition rates of 21.6%, 32.4% and 43.2%, respectively, indicating the limited antibacterial efficacy of H_2_O_2_ and SA/CuHP alone. However, the H_2_O_2_ + SA/CuHP group exhibited enhanced bacterial inhibition (75.7%), surpassing that of H_2_O_2_ and SA/CuHP alone. Correspondingly, the live/dead staining images (Figure 6C) revealed increased dead bacteria (red) in the H_2_O_2_ + SA/CuHP group compared to other groups, indicating enhanced antibacterial efficiency. Furthermore, bacterial morphology was examined via SEM (Figure 6D). A smooth and complete short-rod-shaped surface of bacteria was observed in the Control and SA groups. Minor distortions and wrinkling on the bacterial surface were detected in the H_2_O_2_ and H_2_O_2_ + SA groups, suggesting slight impairments in the bacterial cell membrane’s integrity. When treated with SA/CuHP, bacteria assumed irregular forms and exhibited damaged membranes, whereas the bacteria in the H_2_O_2_ + SA/CuHP group endured more severe shrinkage and cell membrane rupture.

These results suggested that the SA/CuHP hydrogel exhibited exceptional bactericidal capabilities, which were notably enhanced in the presence of H_2_O_2_. The predominant antibacterial mechanism appears to be the synergistic effect of the copper ions combined with the POD-like catalytic properties of the SA/CuHP hydrogel. These findings are consistent with prior research. For example, Gao *et al*. synthesized a gel infused with copper nanodots (Cu-NDs), which displayed significant antibacterial properties attributed to the peroxidase-like activity of the Cu-NDs under acidic conditions [36]. The POD-like activity catalyses a Fenton-like reaction in acidic conditions, transforming H_2_O_2_ into highly toxic •OH [27]. Both •OH and copper ions are known to disrupt the bacterial cell membrane, interfere with intracellular DNA and proteins and ultimately lead to bacterial cell death [80, 81]. In previous studies, some enzymatic materials exhibited satisfactory antibacterial efficacy when combined with photodynamic or photothermal therapy, whereas their inhibition rate without light irrigation was lower than that of the current system [82–84]. Such light-dependent strategies face inherent limitations, including shallow tissue penetration and thermal damage risks [85]. In contrast, the SA/CuHP hydrogel developed in this study leveraged the endogenous H_2_O_2_-rich microenvironment to achieve efficient antibacterial activity without external energy input, demonstrating superior safety and efficacy.

### Establishment of a DP model in rats

**Figure 7.**
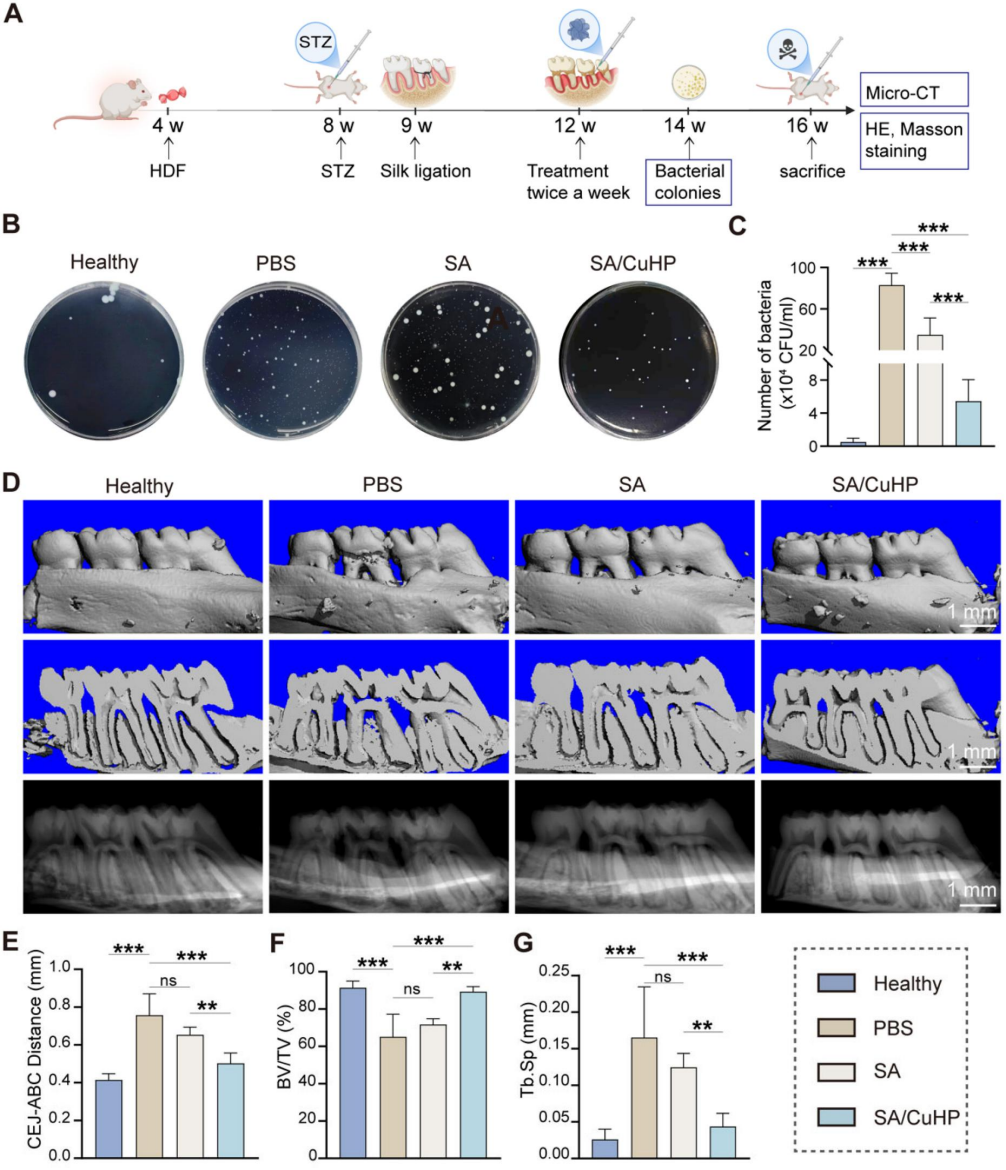
Antibacterial effects and alveolar bone repair properties in rats with DP. (**A**) Treatment schedule. The scheme was created using Biorender.com. (**B**, **C**) RIs of bacterial colonies in gingival fluid from the periodontal pocket and statistical analysis of colony counts. (**D**) RIs of 3D reconstruction, buccopalatal sectional and x-ray of maxillary molars and alveolar bone obtained via micro-CT scanning. Quantification of CEJ-ABC distance (**E**), bone volume per tissue volume (**F**) and trabecular separation (**G**). Data are presented as mean ± SD (*n* = 6).

As depicted in schematic Figure 7A, a model of periodontitis in type 2 diabetic rats was established. The *in vivo* therapeutic efficacy of the SA/CuHP hydrogel was evaluated, focusing on its antibacterial properties, capability for periodontal regeneration and safety. Supplementary Figure S7 shows a persistent blood glucose levels above 16.7 mmol/l in diabetic rats at Days 3 and 7 post-STZ injection, confirming the successful establishment of type 2 diabetes in these rats. These diabetic rats were subsequently utilized to develop experimental periodontitis in the left maxillary M2.

### Retention and degradation *in vivo*

As shown in Supplementary Figure S8, a significant amount of blue hydrogel was present in the periodontal pockets, along the gingival margins and in the palatal regions post-injection. By the first day, residual hydrogel remained within the periodontal pockets; however, by the second day, only minimal traces were detectable near the M2, and by the third day, no discernible retention was observed. These observations suggested that the hydrogel was retained in the periodontal pockets for approximately two days. The limited periodontal retention of SA/CuHP hydrogel primarily stems from its poor adhesion properties, rendering it susceptible to displacement in the wet and dynamic oral environment [86].

Degradation is a critical factor for the efficacious application of hydrogels *in vivo* [87]. Optical imaging revealed a progressive collapse of the hydrogels over a period of 7 days (Supplementary Figure S9A). Quantitative analysis indicated that the SA/CuHP hydrogel had undergone a degradation of 23.7% after three days, increasing to 36.7% after seven days, respectively (Supplementary Figure S9B). The controlled degradation of the composite hydrogel mitigates risks of excessive copper accumulation, thereby preventing copper-induced cytotoxicity in surrounding tissues [88].

### Therapeutic efficacy *in vivo*

The *in vivo* antibacterial properties of the SA/CuHP hydrogel were evaluated using the spread plate method to quantify periodontal bacteria in various groups. Figure 7B and C depicts a considerable presence of bacterial colonies in the PBS group. Notably, treatment with SA/CuHP resulted in a significant reduction of approximately 75.7% of the bacteria, demonstrating a potent antibacterial effect. Intriguingly, the SA group also exhibited a modest antibacterial effect. This outcome could be attributed to the adhesion and retention of the SA hydrogel within the periodontal pocket, which likely facilitated a physical barrier that impeded bacterial invasion.

To evaluate periodontal bone loss in diabetic rats, micro-CT was utilized, and the CEJ-ABC was measured. The 3D reconstructed images and their sectional and X-ray counterparts vividly illustrated significant alveolar bone loss proximal to the ligature around the M2 (Figure 7D). Further measurements of BV/TV and Tb.Sp at the mesial alveolar crest of M2 were conducted to assess bone quality. As depicted in Figure 7E–G, the PBS group displayed increased CEJ-ABC distance and Tb.Sp, along with a decreased BV/TV, clearly indicating substantial bone loss and the successful induction of experimental periodontitis in diabetic rats. In the SA-treated rats, a marginal improvement in bone quality was noted compared to the PBS group, although the differences in CEJ-ABC and other bone parameters did not achieve statistical significance. Crucially, the SA/CuHP treatment significantly ameliorated the induced bone loss, as evidenced by the reduced CEJ-ABC distance and Tb.Sp, along with an increased BV/TV. These micro-CT findings robustly suggested that the SA/CuHP hydrogel exerted a beneficial effect in mitigating bone loss in diabetic rats afflicted with experimental periodontitis.

**Figure 8.**
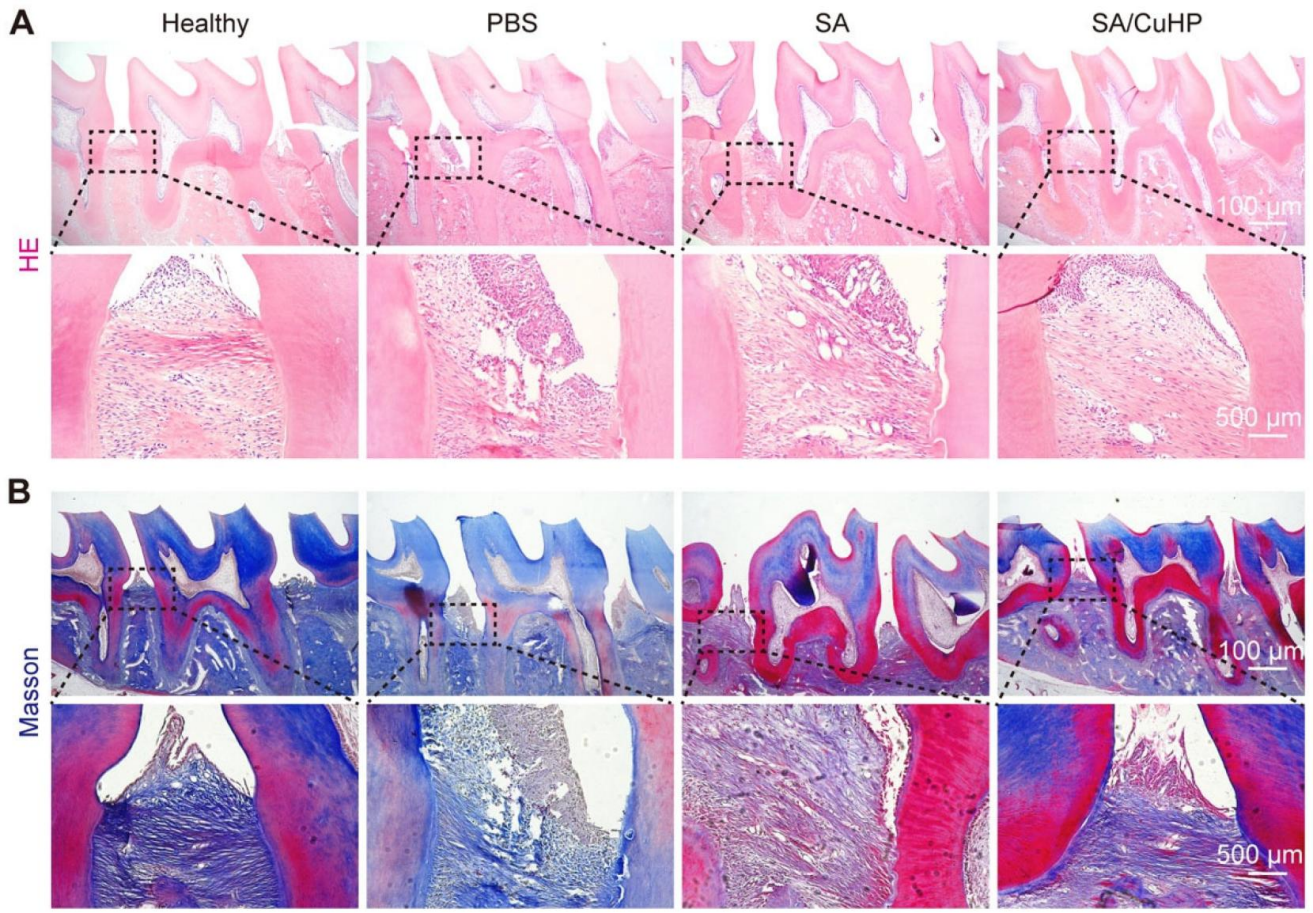
Histological staining of maxillary alveolar bone. (**A**) RIs of HE staining. (**B**) RIs of Masson staining.

To further assess the restoration of damaged periodontal tissue, HE and Masson staining were employed. Compared with the Healthy group, disordered and degraded periodontal ligament fibres around the M2 were observed in both PBS and SA groups. In contrast, the ligaments in the SA/CuHP group exhibited a denser and more regularly organized structure (Figure 8A and B). Overall, these results suggested that SA/CuHP could inhibit periodontal destruction in diabetic rats. Additionally, HE staining of the heart, liver, spleen, lung and kidney revealed no histopathological abnormalities, indicating the *in vivo* safety of SA/CuHP hydrogels (Supplementary Figure S10).

In summary, the SA/CuHP hydrogel demonstrated promising efficacy in the treatment of DP through its mediation of early-stage antibacterial activity and late-phase bone tissue regeneration. This dynamic strategy is promising for addressing the limitations of monofunctional enzyme-mimetic materials, including that antioxidant systems tend to compromise antibacterial efficacy through excessive ROS elimination and pro-oxidative biomaterials risk exacerbating periodontal damage via indiscriminate ROS generation [88, 89]. Despite these promising results, our study is subject to several limitations that necessitate future refinement. First, the poor adhesive properties of the SA/CuHP hydrogel limit its retention and functional durability within the periodontal pocket. Future modifications of the hydrogel composition are planned to enhance its adhesion. Second, this study only preliminarily analysed the potential mechanisms by which the SA/CuHP hydrogel promotes osteogenesis under conditions of oxidative stress. Future work will involve validating the regulation of specific signalling pathways, such as PI3K-AKT. Third, current antibacterial evaluations were restricted to planktonic bacteria; subsequent studies should assess the biofilm dispersal efficacy of the SA/CuHP hydrogel. Fourth, while the SA/CuHP hydrogel treats DP through a pH-dependent functional transition and has demonstrated excellent antibacterial and osteogenic properties in animal experiments, it’s necessary to prioritize advancing the clinical translation studies of the SA/CuHP hydrogel and further validate its clinical applicability and safety through rigorous clinical trials.

## Conclusion

In this study, we developed a SA/CuHP composite hydrogel characterized by its biocompatibility, antibacterial, antioxidant and osteogenic properties, specifically designed for the treatment of DP. The *in vitro* analyses revealed that under neutral pH conditions, the SA/CuHP hydrogel exhibited CAT-like activity, which conferred upon it the capacity to reduce excessive ROS. This activity significantly mitigated the osteogenic inhibition typically induced by oxidative stress. Conversely, in mildly acidic conditions, the hydrogel predominantly demonstrated POD-like activity, thereby exerting potent antibacterial effects. Additionally, the SA/CuHP hydrogel provided sustained release of bioactive copper ions, synergistically enhancing its dual functionality in promoting osteogenesis and suppressing microbial proliferation. In an experimental model of DP, the SA/CuHP hydrogel not only demonstrated robust antibacterial properties but also showed a significant reduction in alveolar bone loss, alongside confirming its safety profile. Overall, these findings reveal that the innovative SA/CuHP composite hydrogel offers a viable therapeutic strategy for addressing DP.

## Supplementary Material

rbaf065_Supplementary_Data
