# Supplementary material for: A pH-sensitive CuHP composite hydrogel featuring antibacterial, antioxidant and osteogenic properties for treating diabetic periodontitis
Source: Regen Biomater. 2025 Jun 23;12:rbaf065. doi: 10.1093/rb/rbaf065 (PMC12448283; doi:10.1093/rb/rbaf065)
Supplement: rbaf065_Supplementary_Data [file rbaf065_supplementary_data.docx]

**Supporting Information**

**A pH-Sensitive CuHP Composite Hydrogel Featuring Antibacterial, Antioxidant, and Osteogenic Properties for Treating Diabetic Periodontitis**

*Xianwen Lu^1,^* ^ξ^*, Sitong Hu^2, 3,^* ^ξ^*, Zhaowenbin Zhang^2, 4,^* ^ξ^*, Jing Bao^1^, Bangping Cao^1^, Jian Xie^1^, Jiang Chang^2^, Chen Yang^2, 5,^* **, Xiaohong Wang^1,^* **, Jiansheng Su^1,^* *

1. Shanghai Engineering Research Center of Tooth Restoration and Regeneration & Tongji Research Institute of Stomatology & Department of Prosthodontics, Shanghai Tongji Stomatological Hospital and Dental School, Tongji University, Shanghai 200072, China.
2. Zhejiang Engineering Research Center for Tissue Repair Materials, Wenzhou Institute, University of Chinese Academy of Sciences, Wenzhou 325000, China.
3. Department of Orthodontics, School and Hospital of Stomatology, Liaoning Provincial Key Laboratory of Oral Disease, China Medical University, Shenyang 110002, China.
4. College of Biological Science and Medical Engineering, Donghua University, Shanghai, 201620, China.
5. Orthopedic Institute, Department of Orthopaedic Surgery, The First Affiliated Hospital, School of Biology & Basic Medical Sciences, Suzhou Medical College, Soochow University, Suzhou, Jiangsu, 215006, China.

ξ These authors have made equivalent contributions to this work and share the first authorship.

*Corresponding Author: [cryangchen@ucas.ac.cn](mailto:cryangchen@ucas.ac.cn); [xiaohongwang@tongji.edu.cn](mailto:xiaohongwang@tongji.edu.cn); [sjs@tongji.edu.cn](mailto:sjs@tongji.edu.cn).

**
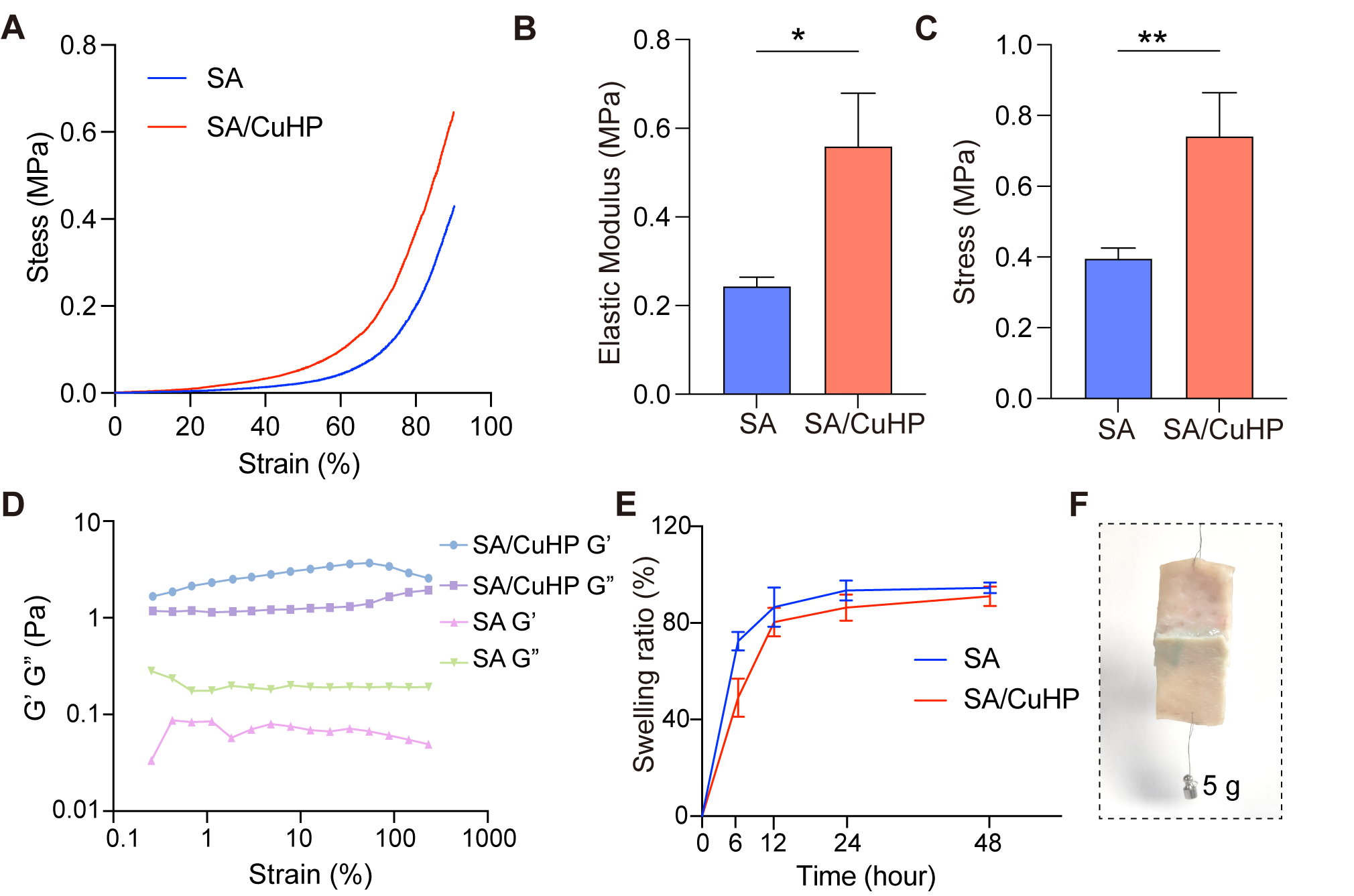
**

**Figure S1.** Characterisation of SA and SA/CuHP hydrogels. (A) The stress-strain curve of the composite hydrogels. (B) The elastic modulus of the composite hydrogels. (C) The maximum stress of the composite hydrogels. (D) The rheological properties of the composite hydrogels. (E) The swelling ratio of the composite hydrogels. (F) The adhesive properties of SA/CuHP hydrogel. Data are presented as mean ± SD (n=3).


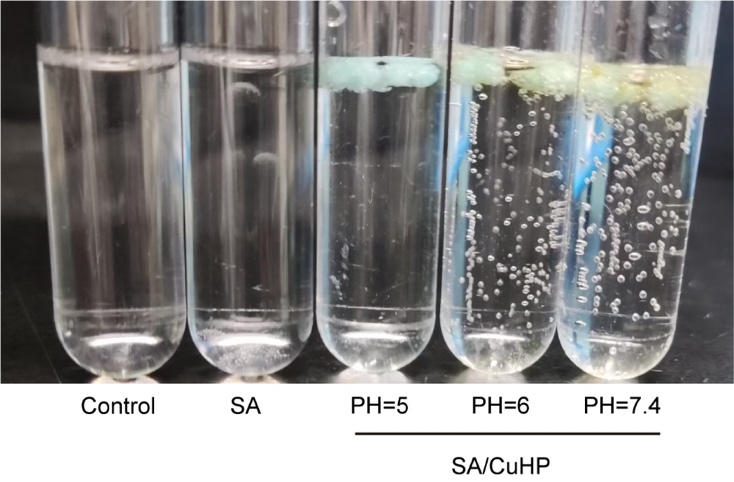


**Figure S2.** Oxygen bubbles production catalyzed by SA/CuHP hydrogel at different pH levels.


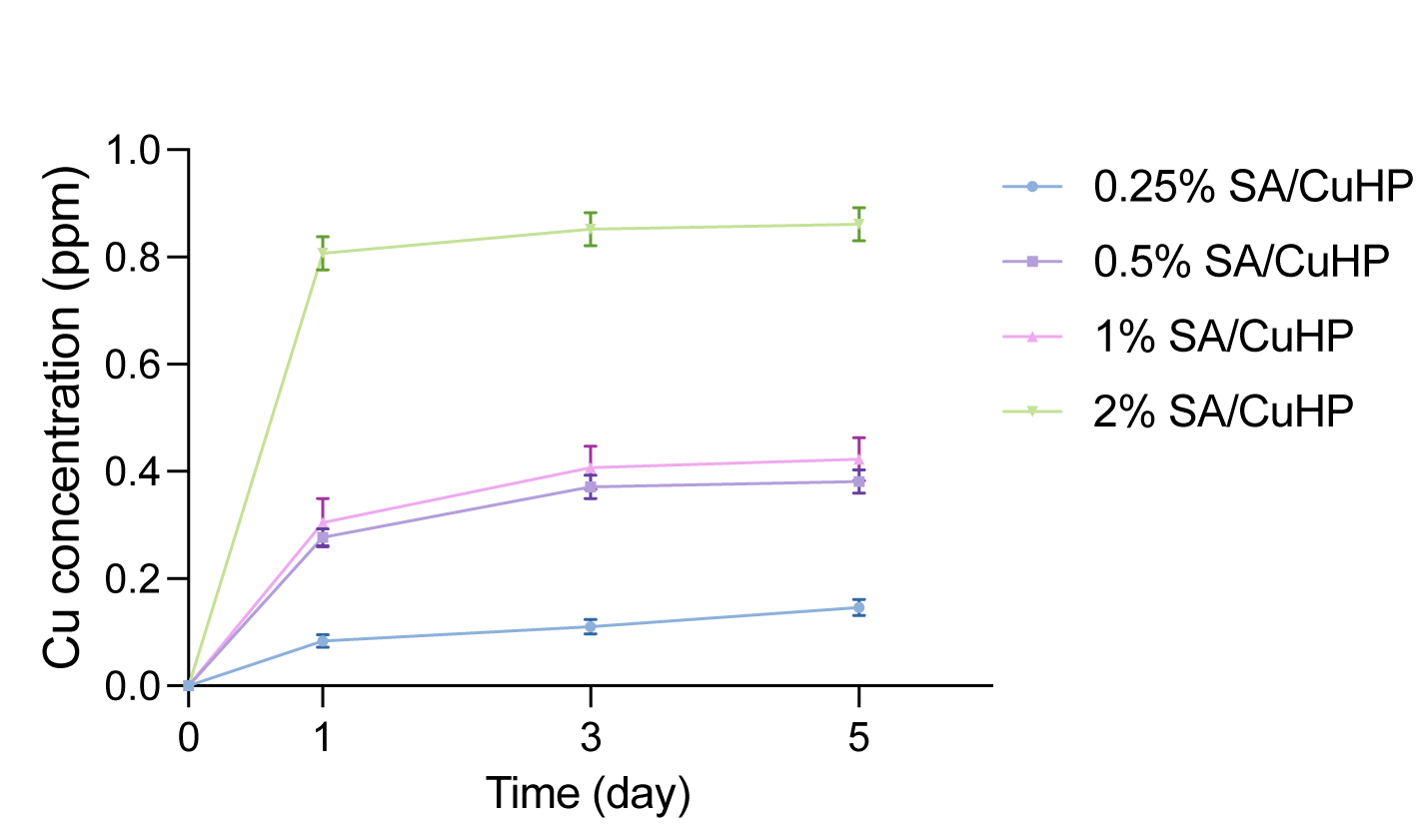


**Figure S3** Accumulated release of copper ions from SA/CuHP hydrogels at different concentrations over 1, 3, and 5 days. Data are presented as mean ± SD (n=3).


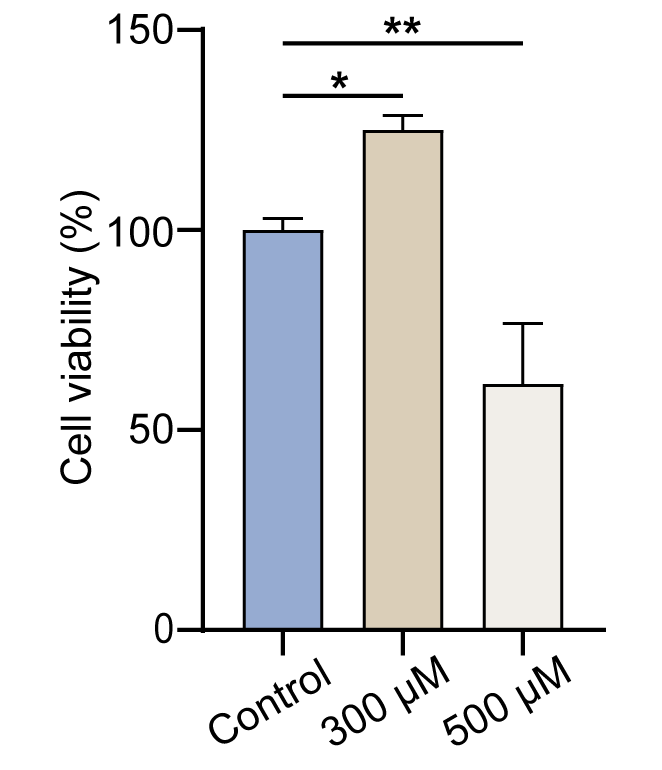


**Figure S4** Establishment of H_2_O_2_-induced oxidative stress models in BMSCs. 300 μM or 500 μM H_2_O_2_ were added into the culture medium for 3 hours, and CCK8 assays were conducted after 24-hour incubation. Data are presented as mean ± SD (n=3).


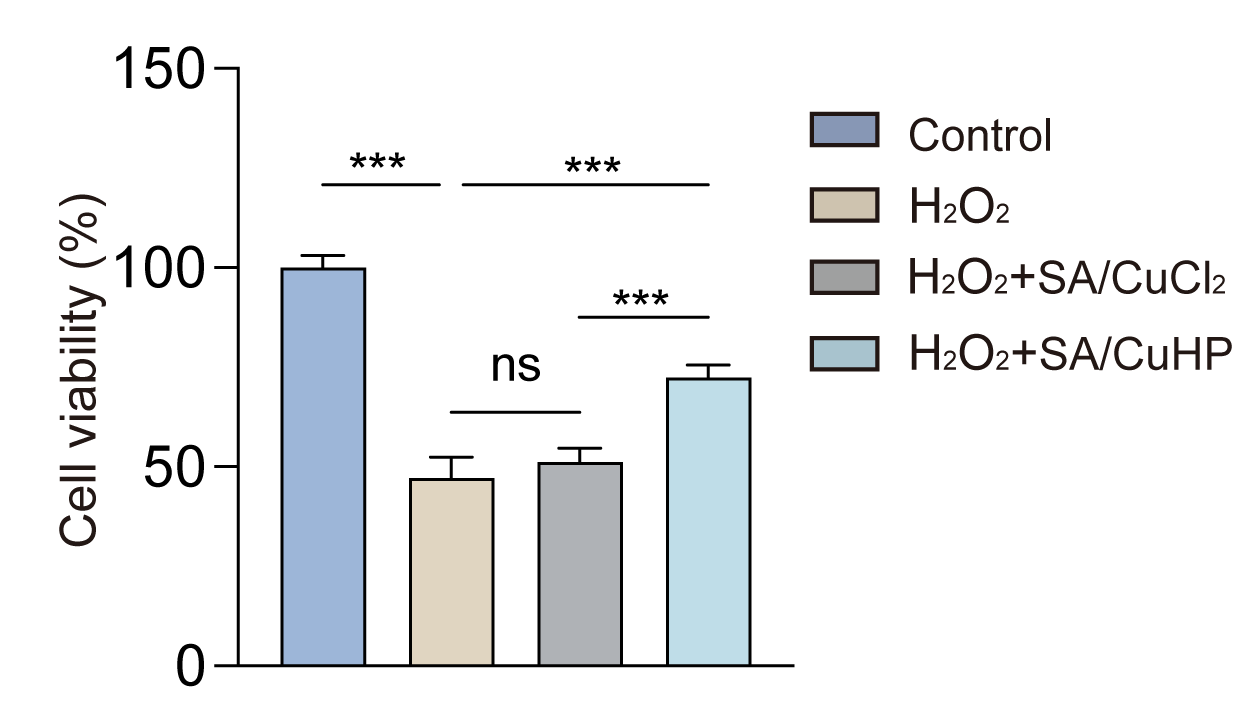


**Figure S5** BMSCs were simulated with different treatment for 3 hours followed by 24-hour culture. Cell viability was measured via CCK8 assay. Data are presented as mean ± SD (n=3).


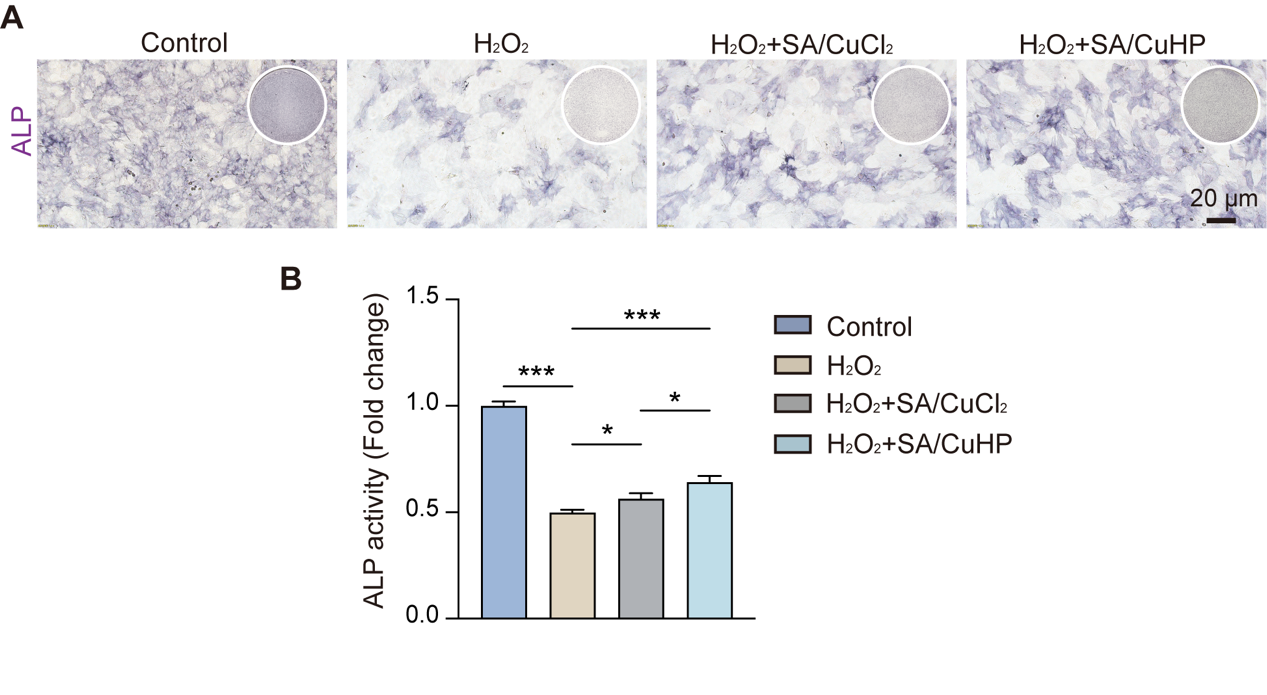


**Figure S6** Osteogenic effects of the composite hydrogels under oxidative stress *in vitro*. (A) Representative ALP staining images of BMSCs with different treatment for 3 hours followed by 7-day culture. (B) Semi-quantitative analysis of ALP activity. Data are presented as mean ± SD (n=3).


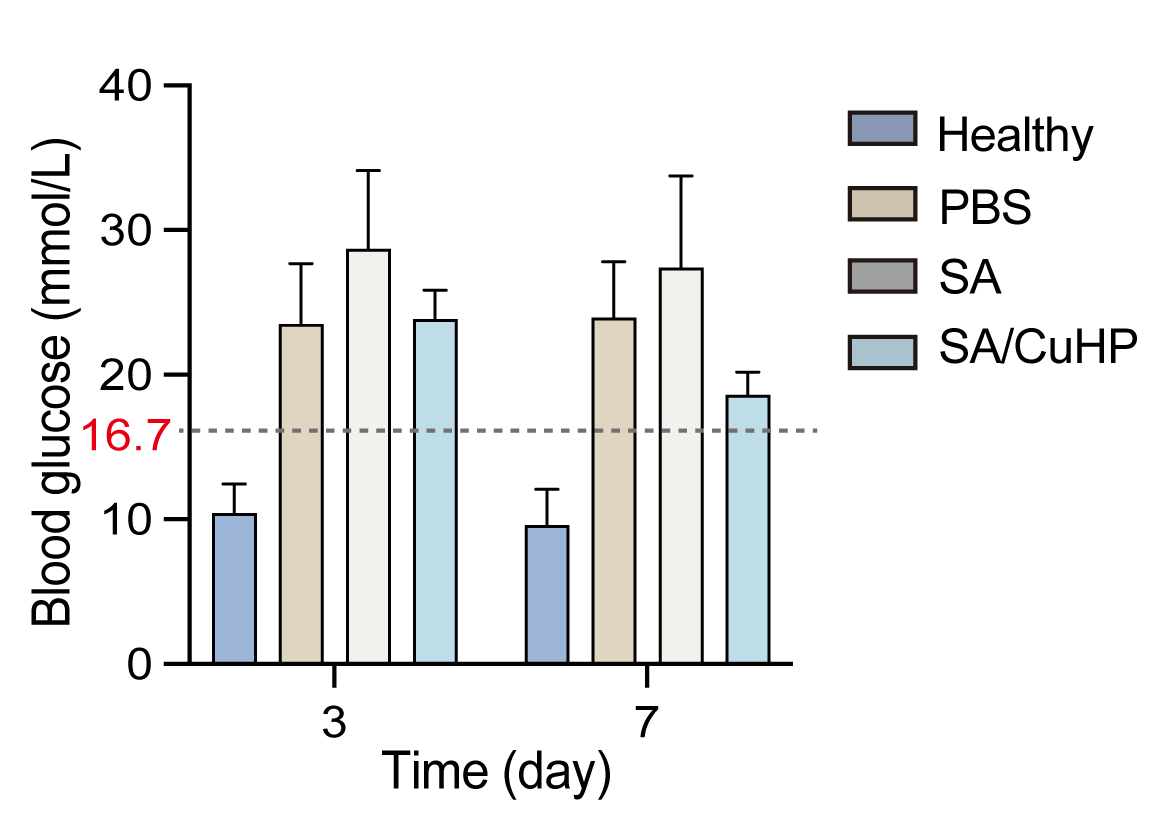


**Figure S7** Blood glucose levels in different groups was measured on day 3 and day 7 after STZ injection. Data are presented as mean ± SD (n=6).


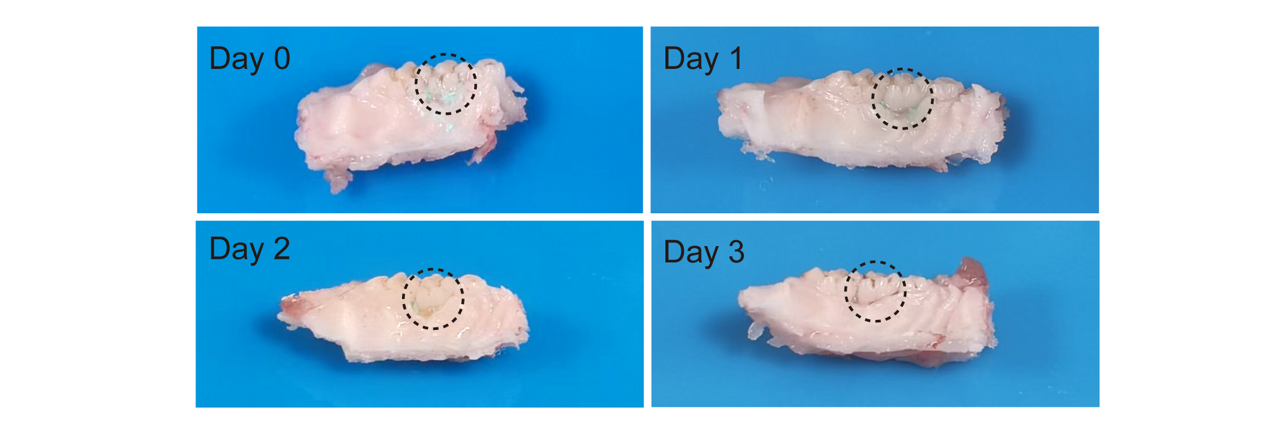


**Figure S8** Retention of SA/CuHP hydrogel *in vivo.*


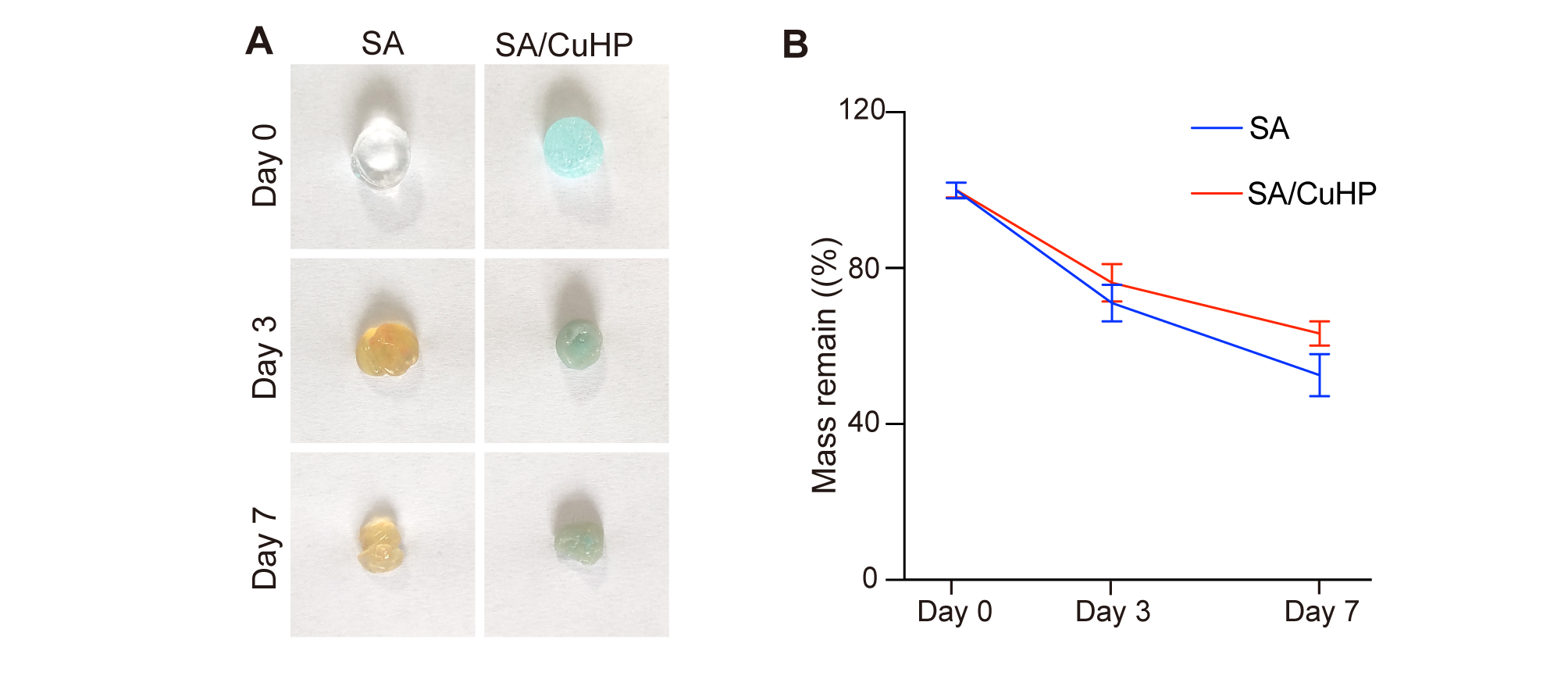


**Figure S9** Degradation of SA and SA/CuHP hydrogels *in vivo*. Data are presented as mean ± SD (n=3).


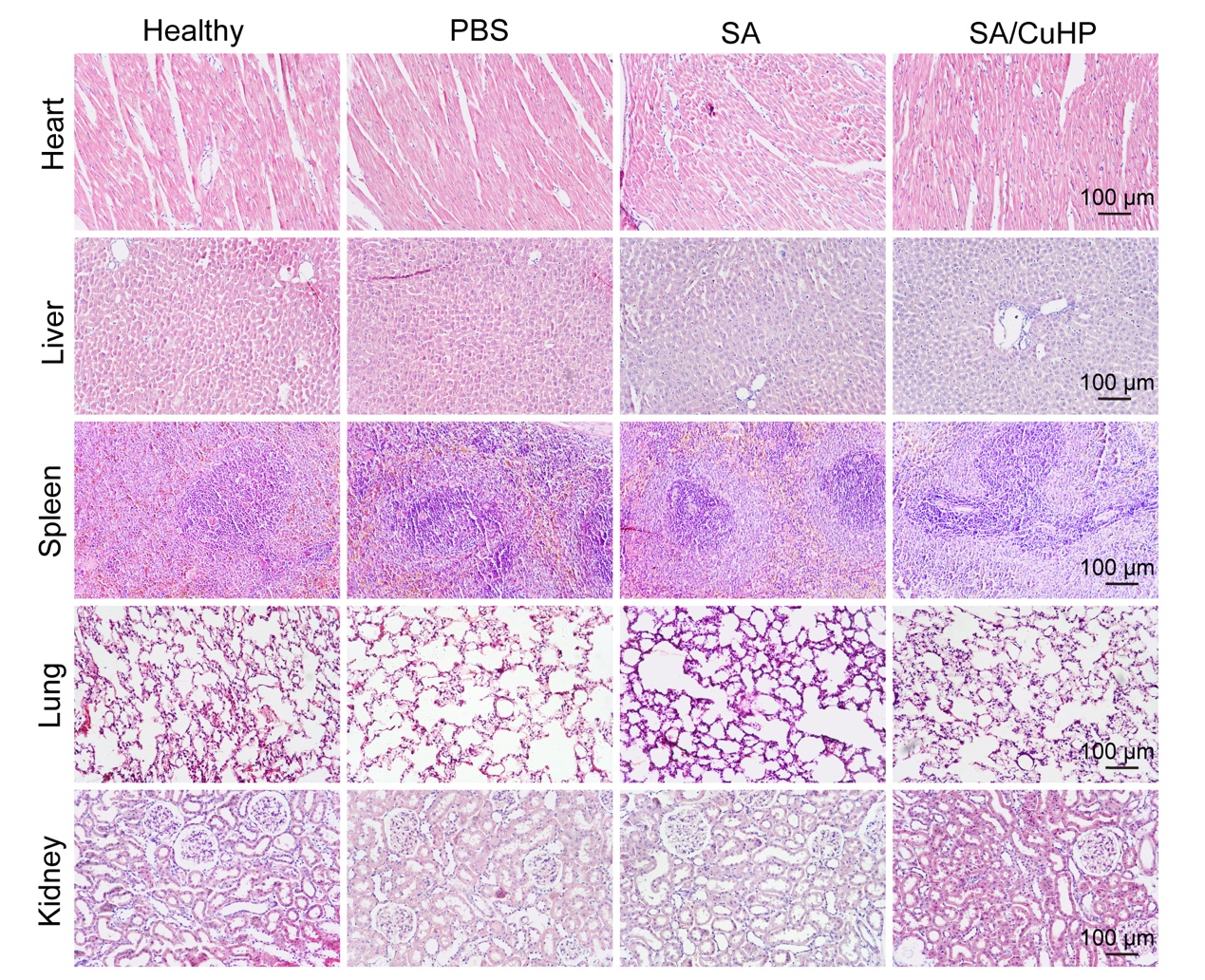


**Figure S10** HE staining of major organs (heart, liver, spleen, lung, and kidney) sections.

**Table S1** Primers sequences for RT-qPCR.

| Gene | Primer sequence (5'-3'） |
| --- | --- |
| *Gapdh(F)* | TCTCTGCTCCTCCCTGTTCT |
| *Gapdh(R)* | TACGGCCAAATCCGTTCACA |
| *ALP(F)* | CCAGAGAAAGAGAGAGACCCC |
| *ALP (R)* | GAGACGCCCATACCATCTCC |
| *OCN(F)* | CTCACTCTGCTGGCCCTGAC |
| *OCN (R)* | CCTTACTGCCCTCCTGCTTG |
| *OSX(F)* | GCCTACTTACCCGTCTGACTTTG |
| *OSX(R)* | TGCCCACTATTGCCAACTGC |
